# LIVING WITH SPINAL CORD INJURY IN THE COMMUNITY OF BANGLADESH: A COMPREHENSIVE ANALYSIS USING THE ICF FRAMEWORK

**DOI:** 10.2340/jrm.v58.44856

**Published:** 2026-06-23

**Authors:** Taslim UDDIN, Mohammad Tariqul ISLAM, Rijwan BHUIYAN, Anika TASNIM, Mohammad Sohrab HOSSAIN, Md Atiqul HAQUE

**Affiliations:** 1 Department of Physical Medicine and Rehabilitation, Bangladesh Medical University, Dhaka; 2 Department of Public Health and Informatics, Bangladesh Medical University, Dhaka; 3 Centre for the Rehabilitation of the Paralysed, Savar, Dhaka, Bangladesh

**Keywords:** activities of daily living, community integration, environment, International Classification of Functioning, Disability and Health, personal autonomy, quality of life, social participation, spinal cord injuries

## Abstract

**Objective:**

To describe limitations in functioning (activities and participation) and environmental and personal factors that are associated with functioning using the ICF framework among persons living with spinal cord injury in the community of Bangladesh.

**Design:**

Cross-sectional study, Bangladesh arm of the International Spinal Cord Injury (InSCI) Community Survey (2023–2024).

**Subjects/Patients:**

789 individuals with spinal cord injury.

**Methods:**

Face-to-face interview through culturally adapted InSCI questionnaires. Generalized linear models were used to determine the associated factors.

**Results:**

Participants were predominantly male (73%), aged 18–40 years (51%), and from rural areas (63%). More than one-third had tetraplegia (34%). Tetraplegic participants reported greater limitations across all ICF domains compared with paraplegics (all *p* < 0.01). Determinants of poorer outcomes included male sex, younger age, low education, rural residence, and severe injury of all ICF domains.

**Conclusion:**

Individuals with spinal cord injury in Bangladesh face severe restrictions in functioning and participation, shaped by sociodemographic and clinical factors. These findings echo global evidence from other InSCI countries and LMIC studies, highlighting systemic barriers common to resource-constrained settings. Addressing these disparities through accessible rehabilitation, policy reforms, and cross-national knowledge exchange is critical to advancing equitable spinal cord injury care worldwide.

## LAY ABSTRACT

Spinal cord injury is a life-changing condition that affects how people move, work, and take part in their communities. In this study, we looked at the everyday challenges faced by people with spinal cord injury in Bangladesh from the launch of the ICF framework. We collected information from nearly 800 participants using a global survey tool that explores health, daily activities, and social participation. Many participants were young men from rural areas, and a large number had severe forms of paralysis. We found that people with more severe injuries experienced greater difficulties in daily life. Sex, age, education, where people live, and severity of injury influenced how well individuals could manage. These findings show that beyond medical care, environmental and social barriers strongly shape the lives of people with spinal cord injury. Improving rehabilitation services and community support can help people live more independent and fulfilling lives.

Spinal cord injury (SCI) represents one of the most disabling health conditions globally, leading to lifelong physical, psychological, and social consequences. The Global Burden of Disease 2019 study estimated that approximately 20.6 million people worldwide are living with SCI, with a disproportionate burden on low- and middle-income countries (LMICs) (1, 2). Bangladesh, a densely populated LMIC, experiences a high incidence of SCI, predominantly due to traumatic causes, often leading to severe neurological impairments (3). Beyond the initial trauma, individuals with SCI often experience persistent impairments, activity limitations, and participation restrictions that significantly affect quality of life and long-term functioning (4, 5). Access to rehabilitation services remains inadequate in many LMICs; more than half of affected individuals do not receive the care they need, thereby limiting recovery and hindering successful reintegration into society (6, 7).

Historically, SCI outcomes have been conceptualized primarily through a biomedical lens, emphasizing neurological level and completeness of injury, secondary complications, and survival (8, 9). Although these parameters remain clinically essential, they insufficiently capture the lived experience of disability in community contexts. Therefore, the World Health Organization, in the International Classification of Functioning, Disability and Health (ICF), articulated a biopsychosocial model that conceptualizes disability as the dynamic interaction between health conditions and contextual factors. The ICF provides a standardized framework that includes body functions and structures, activities and participation, and environmental and personal factors, thereby enabling a holistic and policy-relevant understanding of functioning (10).

Despite the ICF’s widespread conceptual adoption, its empirical application in resource-constrained settings such as Bangladesh remains limited (11). Recent studies from South Asia highlight socioeconomic challenges faced by SCI survivors, including reduced quality of life and unemployment, but often lack a holistic approach to explain the integrated influence of health, environment, and personal factors (5, 12). In Bangladesh, rehabilitation services are largely concentrated in urban tertiary centres, and long-term follow-up is frequently constrained by financial and geographical barriers. Consequently, community-dwelling individuals with SCI experience substantial restrictions in mobility, self-care, employment, and social participation (6, 13).

Understanding functioning within this sociocultural and environmental context is critical for evidence-based rehabilitation planning. The ICF framework enables a multidimensional assessment of functioning by linking impairments, activity limitations, and participation restrictions with environmental facilitators and barriers. Although this approach is highly relevant, comprehensive ICF-based evidence for community-dwelling individuals with SCI in Bangladesh remains scarce. Existing regional literature is primarily focused on hospital-based cohorts, secondary complications, or discrete functional outcomes without integrating core sets or determinants into a unified framework. This gap limits the capacity of policy-makers, clinicians, and rehabilitation planners to design interventions aligned with real-world functioning and participation goals. The Bangladesh component of the International Spinal Cord Injury (InSCI) Community Survey collected comprehensive community-level data using a holistic questionnaire that captures the lived experiences of individuals with SCI, including information aligned with the ICF framework (14). Using this dataset, the present paper aims to describe limitations in functioning (activities and participation) and environmental and personal factors that are associated with functioning using the ICF framework among persons living with SCI in the community of Bangladesh. The findings are expected to generate evidence to inform rehabilitation planning and disability-inclusive policies in Bangladesh and other resource-constrained LMIC settings.

## METHODS

### Study design and participant recruitment

This cross-sectional study was conducted as part of the Bangladesh component of the InSCI Community Survey between January 2023 and June 2024. Bangladeshi individuals aged 18 years or older, who sustained traumatic or non-traumatic SCI and have been living in the community for at least 12 months after injury, were included in this study.

Initially, medical records of approximately 5,000 documented SCI patients from 18 tertiary referral hospitals and 1 specialized rehabilitation centre were screened. From these records, 3,035 individuals with traumatic or non-traumatic SCI or cauda equina syndrome were identified. An additional 436 records were obtained later from other hospitals, including the university SCI centre, as well as individual physicians and surgeons providing SCI care, resulting in a total of 3,471 potentially eligible individuals.

All identified individuals were contacted by telephone, of whom 2,075 were successfully reached. A community survey was subsequently conducted, during which 1,228 individuals completed face-to-face interviews. After excluding 439 individuals who had fully recovered or had incomplete data, a final sample of 789 participants with SCI were included in the present analysis. The participant recruitment process is illustrated in Fig. S1. The methodological procedures of the first phase of the survey have been described previously (3).

### Data collection process

Data were collected through face-to-face interviews using the standardized questionnaire developed for the InSCI Community Survey (14, 15) with country-specific adaptation for Bangladesh (Appendix S1). The questionnaire was translated into Bangla, the primary language of Bangladesh. Trained field interviewers collected data using tablet devices equipped with the Research Electronic Data Capture system to facilitate real-time data entry and ensure accuracy and completeness.

### Instruments and measurements

Sociodemographic and clinical characteristics included sex, age, marital status, education level, current residence, cause of injury, duration of injury, and level and extent of injury. To describe functioning and contextual factors using the ICF framework, variables corresponding to 4 domains were selected based on the ICF coding structure specified in the InSCI study protocol: (a) body functions and structures, (b) activities and participation, (c) environmental factors, and (d) personal factors (14).

*Body functions and structures.* Body functions were assessed to evaluate the level of independence commonly experienced by persons with SCI. The following ICF body function components were included: energy and drive functions (b130), sleep functions (b134), emotional functions (b152), sensation of pain (b280), exercise tolerance functions (b455), defecation functions (b525), urination functions (b620), sexual functions (b640), mobility of joint functions (b710), and muscle tone functions (b735). Each item was rated on a scale ranging from “extreme problem” to “no problem.”. Body structures were represented by the level and completeness of spinal cord lesion (s120) (14).

*Activities and participation.* Activities and participation were assessed based on the level of difficulties experienced in performing daily activities and participating in social roles. The following ICF categories were included: carrying out daily routine (d230), handling stress (d240), changing basic body position (d410), maintaining a body position (d415), transferring oneself (d420), hand and arm use (d445), walking (d450), moving around (d455), moving around using equipment (d465), using transportation (d470), washing oneself (d510), caring for body parts (d520), toileting (d530), dressing (d540), eating (d550), looking after one’s health (d570), doing housework (d640), assisting others (d660), basic interpersonal interactions (d710), intimate relationships (d770), and recreation and leisure (d920). Each item was recorded from extreme problem to no problem. Each item was rated on a scale ranging from “extreme problem” to “no problem” (14).

*Environmental factors.* Environmental factors were assessed to determine the extent to which various environmental contexts made life more difficult. The following ICF categories were included: products and substances for personal consumption (e110), products and technology for personal use in daily living (e115), products and technology for personal indoor and outdoor mobility and transportation (e120), products and technology for communication (e125), products and technology for employment (e135), design and construction of buildings for public use (e150), design and construction of buildings for private use (e155), assets (e165), climate (e225), immediate family (e310), friends (e320), personal care providers and assistants (e340), individual attitudes of acquaintances, peers, colleagues, neighbours, and community members (e425), individual attitudes of health professionals (e450), societal attitudes (e460), social security services, systems and policies (e570), and health services, systems, and policies (e580). Each item was rated from “a lot harder” to “not applicable” (14).

*Personal factors.* Personal factors were assessed using items that evaluated individuals’ self-confidence in performing specific activities and fulfilling social roles. Each item was rated on a 5-point scale ranging from not at all confident to completely confident. These items reflect personal characteristics, beliefs, motivations, and perceived capacity to perform tasks within one’s social and physical environment (15).

### Use of validated tools

Four validated instruments were also used to quantify the status of individuals with SCI. Disability status was evaluated using the Brief Model Disability Survey (Brief-MDS) (16). Functional independence was assessed by using the modified Spinal Cord Independence Measure Self-Report (m-SCIM-SR) (17). Environmental barriers were assessed using the Nottwil Environmental Factors Inventory Short Form (NEFI-SF) (18) and personal factors were evaluated using a self-confidence scale (15). Although these are established, standardized tools, they have not been formally validated in the Bangladeshi context. They were translated into Bangla, pre-tested, and refined based on feedback from data collectors to ensure clarity and contextual relevance.

### Statistical analysis

Sociodemographic and clinical characteristics of the participants were summarized using frequencies and percentages. The distributions of body functions, activities and participation, environmental factors, and personal factors were illustrated using 100% stacked bar charts stratified by injury type (paraplegia and tetraplegia).

For the validated scales, total scores were calculated according to established scoring procedures. The brief-MDS score ranged from 15 to 75, the m-SCIM-SR score ranged from 0 to 100, the NEFI-SF score ranged from 0 to 100, and the personal factors scale ranged from 15 to 45. Differences in scale scores between the paraplegia and tetraplegia groups were examined using the Mann–Whitney *U* test and visualized using box plots.

To identify factors associated with disability status (MDS), functional independence (m-SCIM-SR), environmental factors (NEFI-SF), and personal factors, generalized linear models were fitted separately for each outcome. In these models, the dependent variables were the continuous scores of the respective scales. The independent variables included sex (women = 0, men = 1), age (41–85 = 0, 18–40 = 1), marital status (currently married = 0, others = 1), education (secondary or above = 0, below secondary or no education = 1), current residence (urban = 0, rural = 1), cause of injury (non-traumatic = 0, traumatic = 1), injury level and extent (incomplete paraplegia = 0, incomplete tetraplegia = 1, complete paraplegia = 2, complete tetraplegia = 3), and duration of injury (≤ 5 years = 0, ≥ 6 years = 1). All variables were entered simultaneously into the models to adjust for potential confounding effects.

Adjusted beta coefficients (β) with 95% confidence intervals (CI) were reported. A *p*-value < 0.05 was considered statistically significant. Data were cleaned using Microsoft Excel (Microsoft Corp, Redmond, WA, USA) and analysed using SPSS version 26 (IBM Corp, Armonk, NY, USA).

### Ethical consideration and approval

The study was conducted in accordance with the principles of the 1983 Declaration of Helsinki, with an emphasis on respecting participant autonomy and welfare. Informed consent was obtained from all participants before the interviews began. Participants were provided with a clear explanation of the study’s purpose, procedures, and potential risks, and were assured of their right to decline participation or withdraw at any time without consequence.

To protect privacy, all data were de-identified, securely stored, and accessible only to authorized researchers. Interviewers were trained to maintain strict confidentiality throughout the data collection process. The study involved no invasive procedures and posed no potential health risks to participants. No incentives were provided for participation.

Ethical approval was obtained from the Institutional Review Board of Bangladesh Medical University (formerly Bangabandhu Sheikh Mujib Medical University), Dhaka, Bangladesh (reference no.: 2021-12826). Additional permissions were granted by hospital directors and the Directorate General of Health Services of Bangladesh.

## RESULTS

### Participant characteristics

A total of 789 individuals with SCI were included in the analysis. Participants were predominantly male (73%), aged 18–40 years (51%), and from rural areas (63%). The mean (standard deviation) age was 41.3 (15.0) years. Paraplegia was more common than tetraplegia, accounting for approximately two-thirds of cases (66%), and incomplete lesions were more frequent (74%) than complete lesions. Traumatic causes accounted for 62% of the injuries. The median (interquartile range) duration of injury was 3 (2–4) years. Most participants were currently married (77%). Approximately half of the participants had no formal education or had completed only primary education (Table I).

**Table I:** Background and clinical information on persons with spinal cord injury, Bangladesh (*n* = 789)

Background and clinical information	Overall (*n* = 789) *n* (%)	Paraplegia (*n* = 518)		Tetraplegia (*n* = 271)	
		Complete (*n* = 139) *n* (%)	Incomplete (*n* = 379) *n* (%)	Complete (*n* = 67) *n* (%)	Incomplete (*n* = 204) *n* (%)
Sex					
Women	216 (27.4)	28 (20.1)	124 (32.7)	10 (14.9)	54 (26.5)
Men	573 (72.6)	111 (79.9)	255 (67.3)	57 (85.1)	150 (73.5)
Age					
18–40 years	402 (51.0)	92 (66.2)	200 (52.8)	33 (49.3)	77 (37.7)
41–85 years	387 (49.0)	47 (33.8)	179 (47.2)	34 (50.7)	127 (62.3)
Marital status					
Currently married	605 (76.7)	93 (66.9)	296 (78.1)	54 (80.6)	162 (79.4)
Others^a^	184 (23.3)	46 (33.1)	83 (21.9)	13 (19.4)	42 (20.6)
Education					
Below secondary/no education	396 (50.2)	66 (47.5)	177 (46.7)	35 (52.2)	118 (57.8)
Secondary or above	393 (49.8)	73 (52.5)	202 (53.3)	32 (47.8)	86 (42.2)
Current residence				
Urban	291 (36.9)	60 (43.2)	148 (39.1)	19 (28.4)	64 (31.4)
Rural	498 (63.1)	79 (56.8)	231 (60.9)	48 (71.6)	140 (68.6)
Cause of injury					
Non-traumatic	299 (37.9)	37 (26.6)	173 (45.6)	16 (23.9)	73 (35.8)
Traumatic	490 (62.1)	102 (73.4)	206 (54.4)	51 (76.1)	131 (64.2)
Injury duration					
Up to 5 years	685 (86.8)	112 (80.6)	336 (88.7)	60 (89.6)	177 (86.8)
6 or more years	104 (13.2)	27 (19.4)	43 (11.3)	7 (10.4)	27 (13.2)

a Unmarried/widowed/divorced/separated.

### Body functions and structures

Participants with paraplegia and tetraplegia reported varying degrees of difficulty across physiological and psychological body functions. Most experienced moderate to extreme problems with muscle tone, joint mobility, sensation of pain, and sleep, indicating substantial physiological challenges. In contrast, protective functions of the skin were the only component in which most individuals with tetraplegia reported rarely having no problems. A similar pattern was observed among individuals with paraplegia, for whom protective functions of the skin and shortness of breath were the components most reported as causing rare to no problems.

**Fig. 1:**
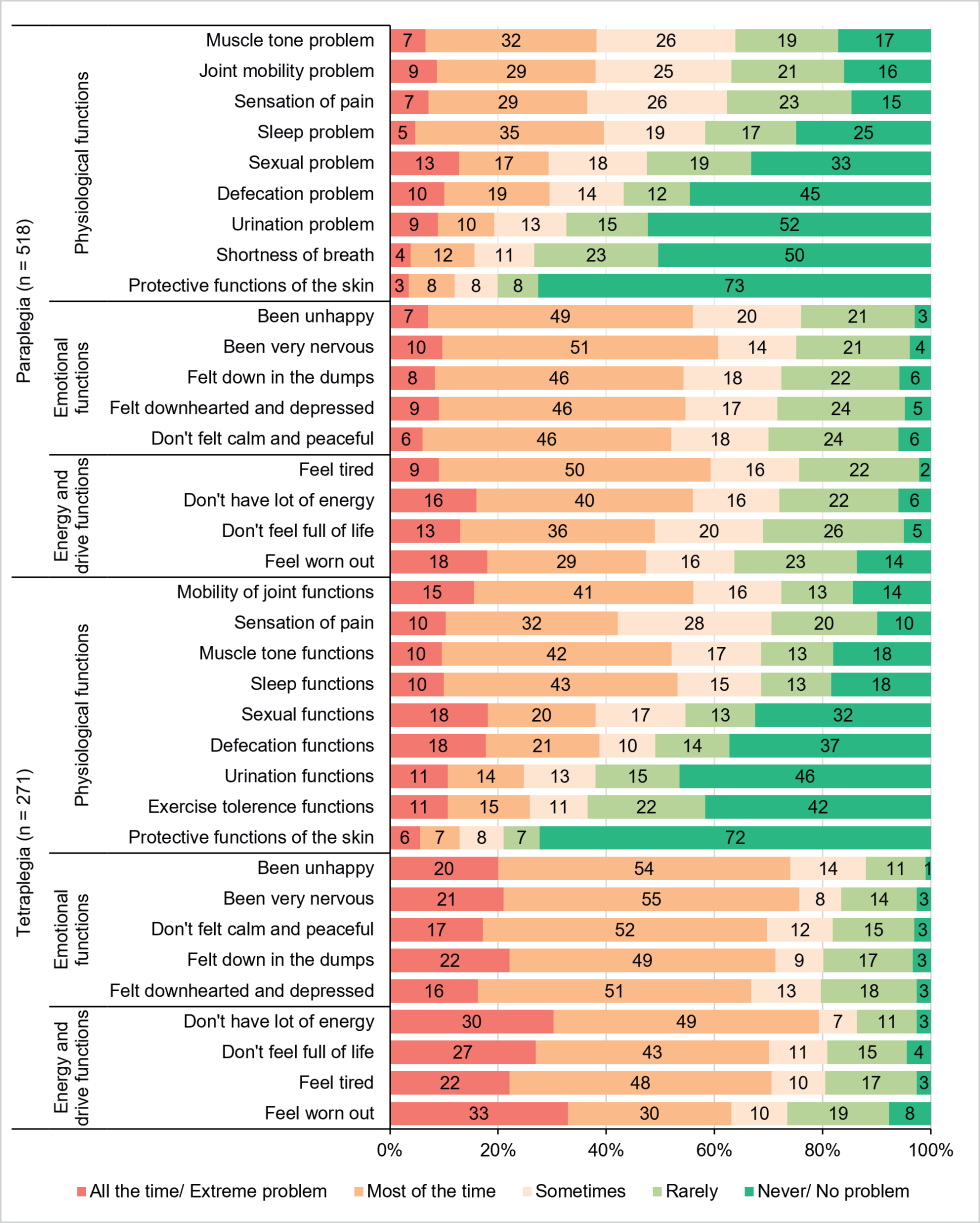
Status of body function of persons with paraplegic and tetraplegic spinal cord injury, Bangladesh (*n* = 789).

Regarding emotional functions, most participants in both groups reported expressing feeling unhappy, nervous, downhearted or depressed, down in the dumps, and not feeling calm or peaceful, ranging from sometimes to all the time. Similarly, limitations in energy and drive functions were prominent. The vast majority of individuals with tetraplegia reported experiencing lack of energy, not feeling full of life, fatigue, and feeling worn out sometimes to all the time. Among individuals with paraplegia, feeling tired and lacking energy were the most commonly reported problems occurring sometimes to all the time. Overall, individuals with tetraplegia reported more body function problems than paraplegia (Fig. 1). For body structure, 48% of participants had incomplete paraplegia, 26% had incomplete tetraplegia, 18% had complete paraplegia, and 8% had complete tetraplegia.

### Activities and participation

Nearly all participants with paraplegia and tetraplegia experienced at least some difficulties in activities and participation. Moderate to extreme difficulties shared by both groups included standing unsupported, carrying out daily routines, performing housework, and managing stress, indicating substantial restrictions in mobility, self-care, and psychosocial functioning. In addition, tasks requiring postural control and transfers such as getting up from lying down and moving around were particularly challenging for individuals with tetraplegia.

**Fig. 2:**
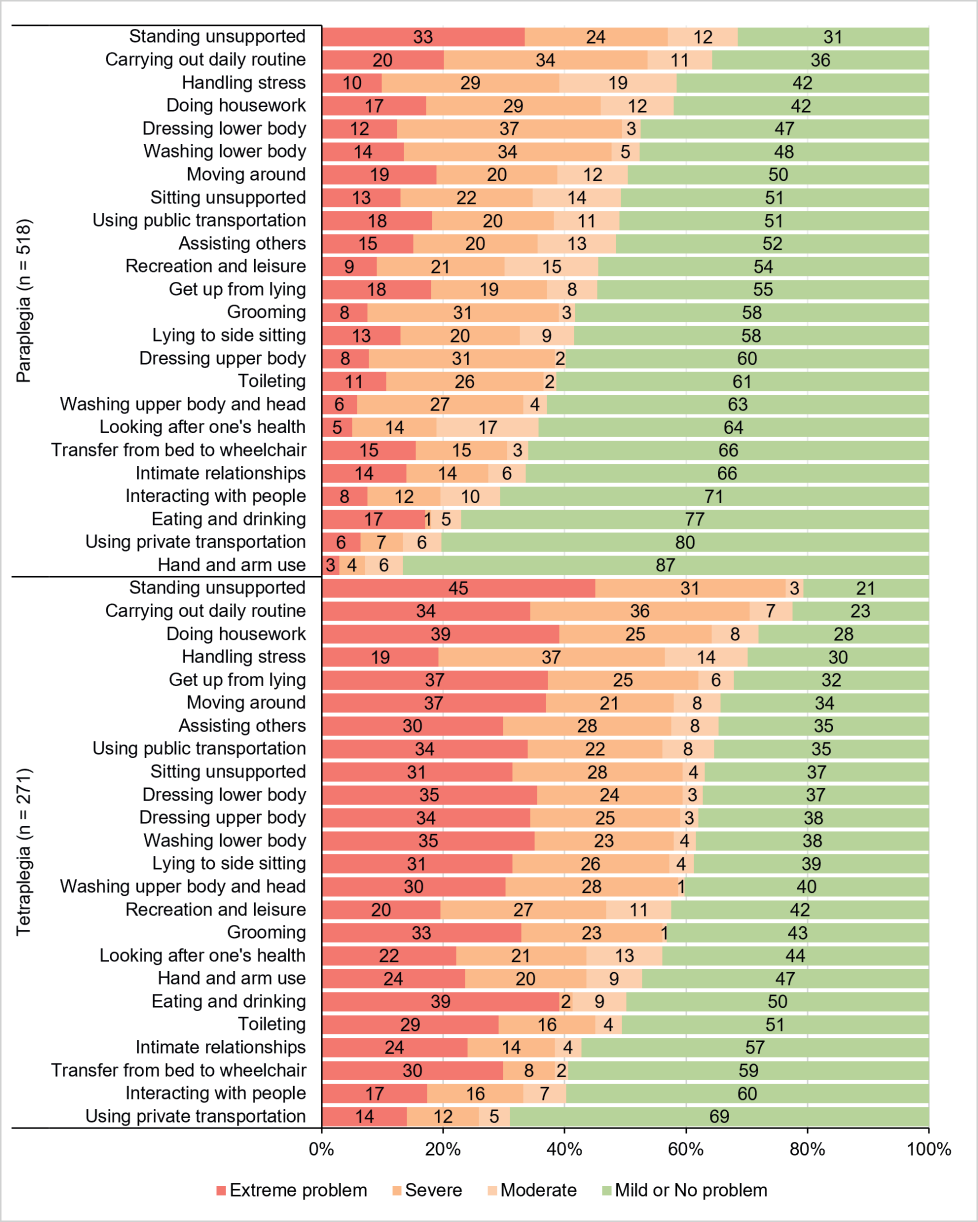
Status of activities and participation of persons with paraplegic and tetraplegic spinal cord injury, Bangladesh (*n* = 789).

In contrast, basic self-care and interpersonal functional activities such as eating and drinking, use of private transportation, and hand and arm use were commonly reported as having mild or no problems, particularly among individuals with paraplegia (Fig. 2). Overall, tetraplegia was associated with more severe and broader participation restrictions.

### Environmental factors

**Fig. 3:**
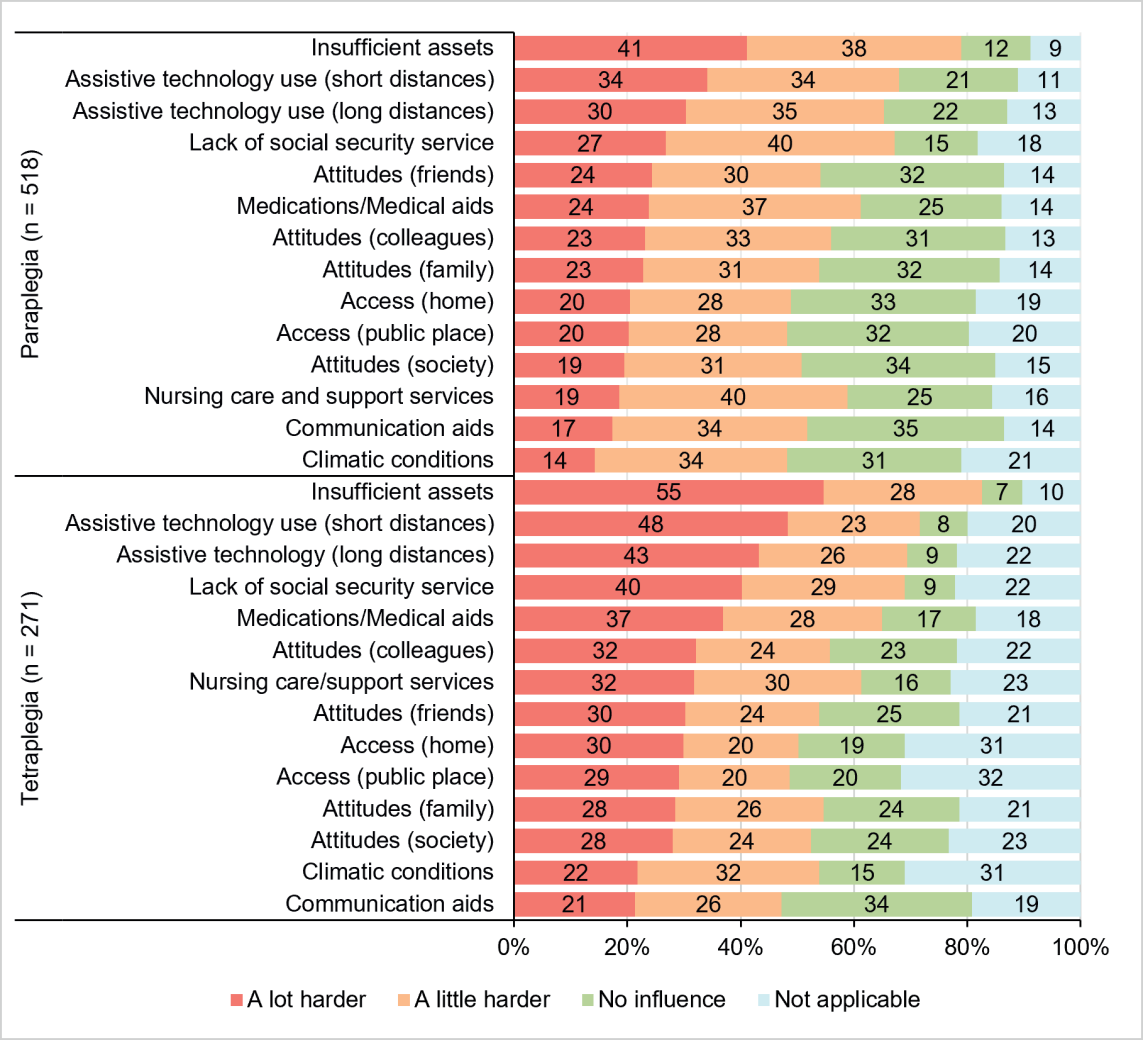
Status of the environmental factors of persons with paraplegic and tetraplegic spinal cord injury, Bangladesh (*n* = 789).

Environmental barriers were commonly reported in both groups, with greater severity observed among individuals with tetraplegia. The most prominent barriers reported that made life “a lot harder” included insufficient assets, limited access to assistive technology for short- and long-distance mobility, and lack of social security services. Additional challenges differed by group: medications and medical aids were notable barriers for tetraplegia, while interpersonal attitudes – particularly from friends – were more frequently reported by participants with paraplegia, with medications/medical aids emerging as a notable barrier among individuals with tetraplegia. Overall, individuals with tetraplegia consistently experienced a higher level of environmental constraints compared with those with paraplegia, highlighting greater vulnerability to contextual barriers in daily living (Fig. 3).

### Personal factors

**Fig. 4:**
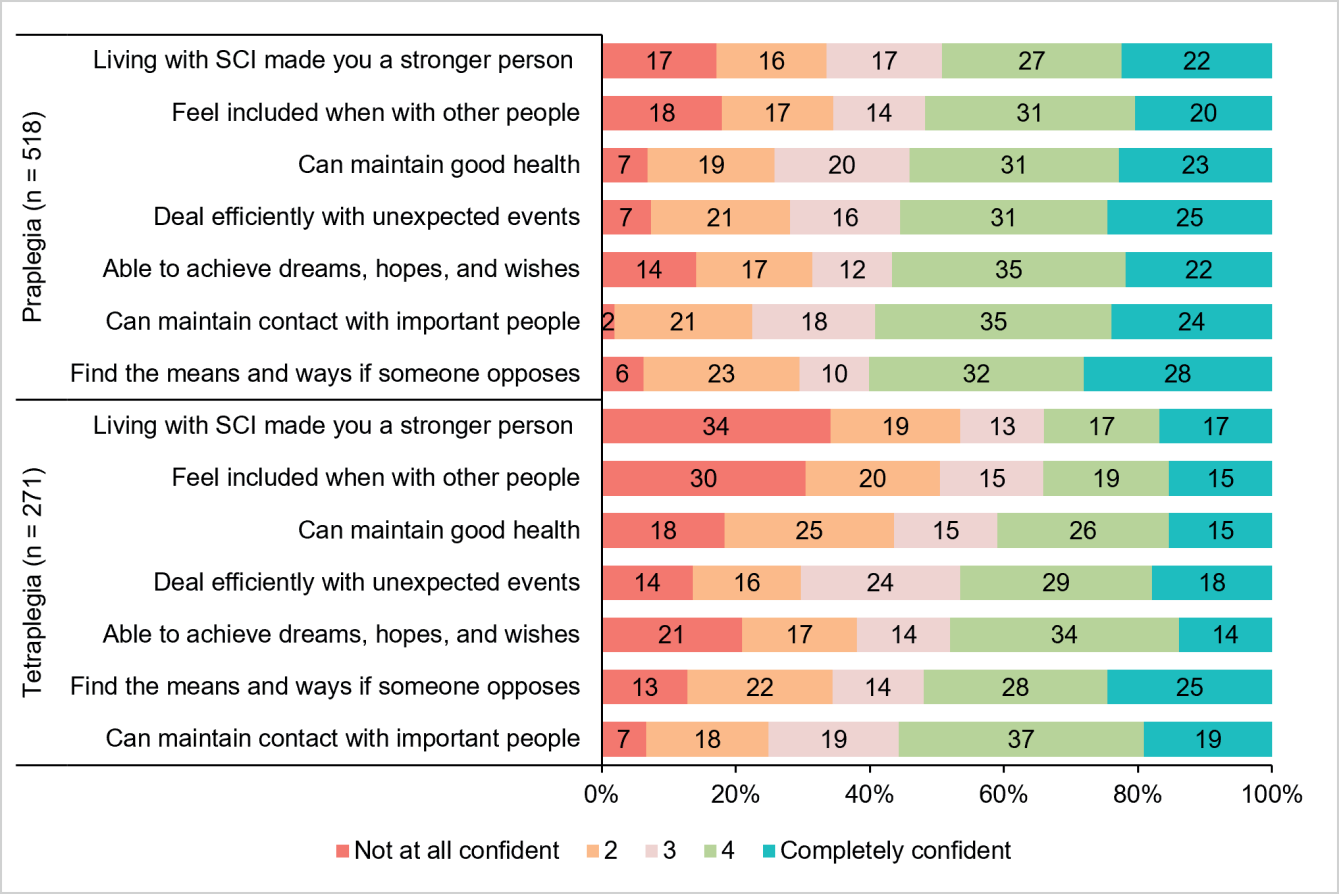
Status of the personal factors of persons with paraplegic and tetraplegic spinal cord injury, Bangladesh (*n* = 789).

Personal factors indicated a comparatively higher level of self-confidence among individuals with SCI. However, individuals with tetraplegia more frequently reported lower levels of confidence (scores 1–2), particularly regarding feeling included among others and perceiving themselves as stronger persons after injury. Across both groups, confidence in maintaining good health appeared comparatively moderate, indicating an area of concern. In contrast, for both groups, a substantial proportion of participants reported being confident (scores 4–5) in maintaining contact with important people and achieving personal goals, with this pattern more pronounced among individuals with paraplegia. Similarly, confidence in handling unexpected events and finding ways to overcome challenges was relatively higher among participants with paraplegia. Overall, these findings suggest that while positive personal perceptions are present, individuals with tetraplegia experience comparatively greater limitations in self-confidence and perceived personal capacity (Fig. 4).

### Factors associated with disability status, functional independence, and environmental and personal factors

The Brief-MDS score of disability status ranged from 0 to 75, a higher score reflecting greater difficulties in activities. The median (interquartile range) score of the Brief-MDS was 39.0 (26.0–54.0). Overall, individuals with tetraplegia reported significantly more difficulties and restrictions compared with those with paraplegia (Fig. S2A). Difficulties and restrictions in activities were significantly associated with having no formal or only primary education, rural residence, and more injury level and extent (Table II).

**Table II:** Factors associated with disability status, functional independence, environmental factors, and personal factors of persons with spinal cord injury, Bangladesh (*n* = 789)

Factors	Brief-MDS score^a^ Score range (0–75)			m-SCIM-SR score^b^ Score range (0–100)			NEFI-SF score^c^ Score range (0–100)			Personal factors score^d^ Score range (7–35)		
	Mean (SD)	β (95% CI)	*p*-value^e^	Mean (SD)	β (95% CI)	*p*-value^e^	Mean (SD)	β (95% CI)	*p*-value^e^	Mean (SD)	β (95% CI)	*p*-value^e^
Sex												
Women	36.7 (15.5)	Ref.		75.2 (25.0)	Ref.		53.0 (27.8)	Ref.		39.7 (12.3)	Ref.	
Men	41.6 (16.2)	2.3 (–0.01, 4.6)	0.051	65.2 (27.4)	–4.8 (–8.3, –1.4)	**0.006**	58.7 (30.0)	4.0 (–0.6, 8.5)	0.091	37.4 (12.5)	–1.4 (–3.3, 0.6)	0.168
Age												
41–85 years	40.7 (15.9)	Ref.		69.1 (25.8)	Ref.		55.0 (29.0)	Ref.		36.4 (12.3)	Ref.	
18–40 years	39.8 (16.4)	–0.9 (–3.1, 1.3)	0.440	66.7 (28.4)	0.2 (–3.1, 3.5)	0.880	59.2 (29.9)	4.2 (–0.1, 8.6)	0.057	39.7 (12.4)	2.8 (1.0, 4.6)	**0.003**
Marital status												
Currently married	39.9 (16.3)	Ref.		68.9 (26.9)	Ref.		55.7 (29.7)	Ref.		37.7 (12.5)	Ref.	
Others^f^	41.3 (15.9)	2.2 (–0.3, 4.7)	0.086	64.5 (27.8)	–3.0 (–6.7, 0.8)	0.118	61.8 (28.5)	4.5 (–0.5, 9.4)	0.077	39.1 (12.3)	–0.7 (–2.7, 1.4)	0.543
Education												
Secondary or above	38.6 (15.9)	Ref.		68.9 (27.0)	Ref.		54.2 (29.9)	Ref.		39.0 (11.8)	Ref.	
Below secondary^g^	41.8 (16.3)	2.6 (0.5, 4.6)	**0.015**	66.9 (26.4)	–2.7 (–5.8, 0.4)	0.085	60.1 (28.9)	7.2 (3.1, 11.3)	**0.001**	37.1 (13.0)	–0.8 (–2.5, 1.0)	0.397
Current residence												
Urban	38.0 (15.6)	Ref.		67.5 (27.5)	Ref.		56.2 (28.4)	Ref.		40.5 (11.8)	Ref.	
Rural	41.5 (16.4)	2.5 (0.3, 4.6)	**0.023**	68.2 (27.0)	1.4 (–1.8, 4.6)	0.385	57.7 (30.2)	0.8 (–3.3, 5.0)	0.693	36.7 (12.6)	–2.9 (–4.6, –1.1)	**0.001**
Cause of injury												
Non-traumatic	37.4 (15.5)	Ref.		73.7 (24.1)	Ref.		54.6 (28.9)	Ref.		39.1 (12.3)	Ref.	
Traumatic	42.0 (16.4)	2.1 (–0.02, 4.3)	0.052	64.4 (28.3)	–3.1 (–6.3, 0.1)	0.058	58.7 (29.8)	0.1 (–4.2, 4.3)	0.971	37.4 (12.5)	–1.7 (–3.5, 0.1)	0.066
Injury level and extent												
Incomplete Para	35.0 (13.5)	Ref.		79.6 (20.1)	Ref.		49.9 (27.0)	Ref.		38.1 (11.0)	Ref.	
Incomplete Tetra	40.7 (15.1)	25.9 (22.2, 29.6)	**< 0.001**	73.3 (23.7)	–49.5 (–55.0, –43.9)	**< 0.001**	57.9 (28.7)	18.6 (11.3, 26.0)	**< 0.001**	37.7 (12.4)	–8.2 (–11.3, –5.1)	**< 0.001**
Complete Para	43.1 (16.9)	7.2 (4.4, 10.0)	**< 0.001**	47.0 (22.2)	–30.7 (–34.9, –26.5)	**< 0.001**	69.9 (29.8)	17.9 (12.4, 23.5)	**< 0.001**	42.9 (13.8)	4.9 (2.5, 7.2)	**< 0.001**
Complete Tetra	62.1 (10.9)	4.8 (2.3, 7.1)	**< 0.001**	28.7 (19.3)	–5.4 (–9.1, –1.8)	**0.004**	69.4 (32.6)	7.5 (2.6, 12.3)	**0.002**	28.9 (12.6)	0.5 (–1.5, 2.6)	**0.622**
Injury duration												
Up to 5 years	40.0 (16.4)	Ref.		68.7 (27.3)	Ref.		56.4 (29.7)	Ref.		37.8 (12.5)	Ref.	
6 or more years	41.5 (14.4)	2.3 (–0.7, 5.3)	0.136	62.9 (25.7)	–4.1 (–8.6, 0.4)	0.072	62.3 (28.4)	6.1 (0.2, 12.0)	**0.043**	39.7 (12.0)	0.6 (–1.9, 3.1)	0.622

a Brief Model Disability Survey, higher scores reflect greater difficulties.

b Modified Spinal Cord Independence Measure Self-Report, higher score indicates more independence.

c Nottwil Environmental Factors Inventory Short Form, higher scores indicating greater environmental barriers and challenges.

d Self-confidence in performing specific tasks or roles, higher scores indicating greater self-confidence.

e *P*-value adjusted for all factor variables in the model simultaneously.

f Unmarried/widowed/divorced/separated.

g Including no formal education.

SD indicates standard deviation; CI indicates confidence interval. Items shown in bold are statistically significance.

The m-SCIM-SR score of functional independence ranges from 0 to 100. A higher score indicates more independence. The median (interquartile range) score was 76.3 (44.7–92.1). Individuals with tetraplegia experienced significantly lower functional independence compared with those with paraplegia (Fig. S2B). In the statistical model adjusted for all factor variables, male sex, and more injury level and extent were found to be associated with less functional independence among person with SCI (see Table II).

The NEFI-SF score of environmental barriers ranges from 0 to 100, with higher the scores reflecting the greater barriers. The median (interquartile range) score was 63.0 (36.0–78.0). Participants with tetraplegia reported greater environmental barriers compared with those with paraplegia (Fig. S2C). The presence of environmental barriers was associated with having no formal or only primary education, more injury level and extent, and an injury duration of 6 or more years (Table II).

The personal factors score of less self-confidence in performing specific tasks or roles ranges from 7 to 35. The higher the score reflect the greater the self-confidence. The median (interquartile range) score of personal factors was 22.0 (16.0 30.5). Overall, individuals with tetraplegia reported lower confidence in personal factors compared with those with paraplegia (Fig. S2D). Less confidence in personal factors was associated with younger age (18–40 years), rural residence, and more injury level and extent (see Table II).

## DISCUSSION

The Bangladesh component of the 2023–2024 InSCI survey represents the largest community-based investigation of SCI conducted in the country to date and the first large-scale application of the ICF framework in this population. By comprehensively describing functioning, activity and participation restrictions, and contextual factors, this study provides a detailed account of the lived experiences of individuals with SCI in community settings. The findings are broadly consistent with evidence from other LMICs, highlighting both shared challenges and the amplified burden associated with resource constraints settings (5).

### Multidimensional functioning problem

Findings of the study demonstrate that individuals living with SCI in Bangladesh continue to experience substantial physiological and psychological impairments long after the acute phase of care. Motor-related problems, particularly abnormalities in muscle tone, reduced joint mobility, pain, and sleep disturbances remain highly prevalent, indicating that functional recovery is often incomplete and that secondary complications continue to shape daily life. While these issues are well documented globally (19, 20), their persistence in community settings in LMICs likely reflects limited access to long-term rehabilitation, follow-up care, and preventive services (21).

A considerable psychological burden was also evident. Participants frequently reported symptoms of feelings of unhappiness, nervousness, depressive mood, reduced inner peace, and low energy. These challenges were more pronounced among individuals with tetraplegia, likely due to greater functional dependence, chronic pain, sleep disruption, and socioeconomic stressors. Such findings are consistent with evidence showing that depression, anxiety, and fatigue are common long-term complications of SCI and are strongly associated with reduced life satisfaction (22). In resource-constrained settings, limited access to mental health and community-based support services may further exacerbate these issues (4, 21).

From a clinical and policy perspective, these findings emphasize that living with SCI in the community involves complex, interrelated physiological and psychological functional challenges requiring sustained, multidisciplinary management. Rehabilitation strategies in Bangladesh should therefore extend beyond early motor recovery to include long-term spasticity control, pain management, sleep care, and routine psychological screening and community-based rehabilitation follow-up. Integration of mental health services, caregiver training, and telerehabilitation may help address long term needs in resource-constrained settings.

### Activities and participation restrictions

Mobility-related limitations emerged as a major barrier to independence and social engagement, particularly among individuals with higher-level injuries. Difficulties in movement, transfers, and balance restrict participation across home and community environments. Similar patterns have been observed in other LMICs, where mobility impairment is a key determinant of reduced autonomy and quality of life after SCI (23).

Participation in domestic and social roles was also notably restricted. Difficulties in performing housework, assisting family members, managing daily responsibilities, and coping with stress suggest that community reintegration does not necessarily translate into meaningful participation. In Bangladesh, these challenges are likely exacerbated by inaccessible public spaces, transportation barriers, and limited vocational rehabilitation opportunities.

These findings highlight the importance of rehabilitation programmes that prioritize functional mobility, transfer skills, and community navigation, alongside psychosocial coping strategies. From a policy perspective, improving accessible transportation, inclusive infrastructure, and vocational reintegration programmes is essential to enhance participation and reduce long-term dependency.

### Environmental barriers

Environmental conditions substantially shape long-term community outcomes after SCI. Economic hardship, limited availability of assistive technologies, gaps in social protection services, and restricted access to essential medical supports emerged as major contextual constraints that limited independence and participation. Social attitudes and support systems further influenced daily life functioning, indicating that participation is strongly mediated by the interaction between impairment and the surrounding social and service environment context. Evidence from both LMICs and high-income countries indicates that environmental barriers persist across settings, although their nature and severity differ (24–27). In Bangladesh, structural limitations such as poverty, weak service systems, and inadequate infrastructure likely intensify these barriers and contribute to long-term exclusion.

Addressing these challenges requires integrating environmental considerations into rehabilitation planning. Expanding access to assistive devices, strengthening social protection mechanisms, improving transport and built environments, and scaling up community-based rehabilitation (CBR) services are critical. Policies promoting universal accessibility and inclusive design can substantially reduce participation restrictions and improve quality of life.

### Personal factors

Individuals living with SCI in the community reported moderate self-confidence in relationships and pursuing personal goals. However, confidence was consistently lower among persons with tetraplegia, especially regarding social inclusion and perceiving themselves as stronger after injury. This gradient aligns with international evidence showing that greater injury severity and functional dependence are strongly associated with reduced self-efficacy, perceived control, and psychological adjustment after SCI (28–30).

The moderate confidence in maintaining good health across both groups is clinically significant, because long-term SCI management requires sustained self-management skills, access to rehabilitation follow-up, and supportive community systems. Evidence suggests that self-efficacy is a key determinant of secondary complication prevention, psychological well-being, and quality of life (31, 32). In LMIC contexts, where continuity of care and psychosocial services may be limited, strengthening self-management capacity is essential. Interventions such as peer support, structured education programmes, and community-based rehabilitation initiatives may enhance resilience, confidence, and long-term adaptation.

### Determinants of disability status, independence, environment, and personal factors

Injury severity emerged as the most consistent determinant across all outcomes, including disability, functional independence, environmental barriers, and personal confidence. Individuals with tetraplegia experienced significantly poorer outcomes than those with paraplegia, reflecting greater neurological impairment and dependency. This finding is consistent with global evidence identifying injury level and completeness as key predictors of long-term functioning (33, 34).

Socioeconomic and contextual factors further shaped outcomes. Lower education and rural residence were associated with greater disability and environmental barriers, suggesting that limited health literacy, reduced access to rehabilitation services, and infrastructural disadvantages compound functional limitations. Comparable findings have been observed in LMIC settings, where geographic inequities, weaker service networks, and financial hardship intensify participation restrictions and restrict access to assistive technologies and community resources (2, 35, 36). Longer injury duration was also linked with higher environmental barriers, possibly reflecting cumulative exposure to inaccessible infrastructure, economic strain, and progressive secondary complications. Together, these findings underscore that disability after SCI is not only mediated by biomedical factors but also by social determinants and environmental context.

Functional independence was lower among men after adjustment, although evidence on sex differences remains mixed (37). Younger individuals demonstrating lower personal confidence may reflect disrupted life trajectories, employment uncertainty, and social reintegration challenges, a pattern also documented in longitudinal SCI cohorts (22). These findings emphasize the need for targeted rehabilitation strategies that prioritize individuals with severe injuries, limited education, and rural residence. Strengthening CBR programmes, improving access to assistive technologies, and addressing structural inequities are essential to reduce disparities in outcomes (38).

### Novel contributions

This study makes 3 key contributions to the global SCI literature. First, it provides one of the largest LMIC datasets (*n* = 789), offering robust evidence for benchmarking against other InSCI countries. Second, the identification of male sex, rural residence, low education, and severe injury as cross-cutting determinants highlights structural disadvantages that may extend across settings, consistent with cross-country findings. Third, the use of the ICF framework enables direct comparison with international data, helping bridge knowledge gaps between LMIC and HIC contexts.

### Policy and clinical implications

The co-occurrence of functional limitations and environmental barriers underscores the need for multilevel interventions. Health systems should strengthen CBR, ensure access to affordable assistive devices, and provide long-term follow-up services. Policy frameworks should integrate disability perspectives into national health and transport planning, with financial support mechanisms for individuals with SCI.

### Limitations and future directions

This study has several limitations. Its cross-sectional design restricts causal inference, and the exclusion of fully recovered individuals may have led to an overestimation of disability. As this study was conducted within the InSCI survey rather than as a dedicated ICF-based investigation, some aspects of functioning and contextual factors were not captured. The findings should therefore be interpreted as a pragmatic, rather than comprehensive, application of the ICF framework.

Data were collected through face-to-face interviews, including instruments originally designed for self-administration. This approach was necessary given the low educational attainment of many participants; however, it may have introduced interviewer and social desirability bias, particularly for subjective measures. Additionally, these instruments have not been formally validated in the Bangladeshi context, which may affect the cultural appropriateness and accuracy of the measurements.

Despite these limitations, the study provides important baseline evidence. Future research should adopt longitudinal designs to examine changes in functioning over time and incorporate qualitative approaches to better understand lived experiences. Expanding the use of comprehensive ICF core sets and culturally adapted measurement tools will further strengthen the validity and policy relevance of findings.

### Conclusion

Individuals with spinal cord injury (SCI) in Bangladesh experience substantial limitations in activities and participation, compounded by environmental barriers and differences in personal factors. Individuals with tetraplegia consistently experienced poorer outcomes across all domains compared with those with paraplegia. Injury severity, sex, age, education, and rural residence were significantly associated with functioning, underscoring the combined influence of biomedical and contextual determinants.

Improving outcomes requires a multidimensional approach that goes beyond clinical care, including expanded access to rehabilitation, strengthened community-based support, improved accessibility, and efforts to reduce socioeconomic inequities. These findings align with global evidence and highlight the need for context-specific, yet globally informed, strategies to advance equitable and inclusive SCI care.

## Supplementary Material




