# Supplementary material for: LIVING WITH SPINAL CORD INJURY IN THE COMMUNITY OF BANGLADESH: A COMPREHENSIVE ANALYSIS USING THE ICF FRAMEWORK
Source: J Rehabil Med. 2026 Jun 23;58:44856. doi: 10.2340/jrm.v58.44856 (PMC13309837; doi:10.2340/jrm.v58.44856)
Supplement: Supplementary file 1 [file JRM-58-44856-s1.pdf]

Bangladesh Spinal Cord Injury (InSCI) Community Survey

International Spinal Cord Injury Survey (InSCI)

The second wave of the worldwide survey
on community-dwelling persons with spinal cord injury.

This version April 2022

Countries all over the world take part in this initiative to capture the experience of living with spinal cord injury by asking those who know best: persons with spinal cord injury.

A collaboration of

ISPRM International Society of Physical and Rehabilitation Medicine

ISCoS The International Spinal Cord Society

Dear participant

Welcome to the InSCI survey, we are very happy to have you on board!

InSCI is the first worldwide survey on community-dwelling persons with spinal cord injury and in its' first round in 2017, over 12'500 persons with spinal cord injury from 22 countries participated. It is our pleasure that the InSCI survey goes into the second round to capture the experience of living with spinal cord injury by asking those who know best: persons with spinal cord injury.

Please fill in the questionnaire as completely as possible and don't leave any questions unanswered. There is no right or wrong and no good or bad answer. It is important that you answer spontaneously and decide which response best applies to your personal situation.

You can also complete the questionnaire online at [www.insci.com]. Please login with your InSCI-ID and your personal password:

Your InSCI-ID is: ######

Your personal password is: ######

We guarantee that your data is protected with the highest security standards. No personal data will be handed out to third persons outside the study center. All questionnaires are anonymized by a unique identification number (InSCI-ID) and there is no personal information such as name or address on the paper or online questionnaire.

In case you have any question or need support in questionnaire completion, we are happy to help. Please send us an email at contact@en.insci.network or contact our toll-free InSCI-helpline at 0700 523 696 631.

Thank you again for your commitment!

Your InSCI-Team

Personal information

1. **Are you?**

- ○ Male
- ○ Female
- ○ Other

2. **What is your date of birth? Age?**

DD / MM / YYYY   Age
□□/□□/□□□□   ✎...........

3. **In which country were you born?**

✎..............................

4. **What is your city/province of residence and postal (ZIP) code if available? Indicate if it is an urban or rural area:**

City<br>**District /Division**<br>ZIP code<br>Urban/rural

5. **What is your current marital status?**

- ○ Single
- ○ Married
- ○ Cohabiting or in a partnership
- ○ Separated or divorced
- ○ Widowed

6. **Who lives in your household with you?**

Check all that apply

- □ I live alone
- □ Children under 14 years of age, number: ✎...............
- □ Youth between 14 and 18 years of age, number: ✎................
- □ Persons between 18 and 64 years of age, number: ✎................
- □ Persons over 64 years of age, number: ✎................
- □ I live in an institution *e.g. home for the elderly, nursing home*

7. **Do you get assistance with your day-to-day activities at home or outside?**

- ○ No
- ○ Yes, by the following persons:

  Check all that apply
  - □ Family
  - □ Friends
  - □ Professionals or paid assistants

7.1 **If yes, then where you get the help?**

- □ Get help with housework (eg; eating, bathing)
- □ Get help with outdoor activities (eg, going to the market, shopping)
- □ Get help both at home and abroad

8. **What is the highest level of education that you have completed?**

No schooling	No institutional schooling
Primary	class 1 to class 5(PSC/ ebtedayee)
Lower secondary	class 6 to class 8(JSC/ Junior Dakhil)
Higher secondary	class 9 to 10 (SSC/ Dakhil/Vocational/trade course)
Post-secondary	class 11 to 12 (HSC/Alim)
Short tertiary	BHMS/B.Sc. Nursing, Fine Arts, Music, Unani Medicine & Surgery
Higher education (Bachelor/Master/Dr)	Snatok(Pass/Sanman)/B.Sc. Eng. /Fazil (Sanman/Pass) /Degree (Honours)/B.Ed/B.PEd/MBBS/BDS/Masters/Kamil/M.Ed/MA/M.Sc./PGD/M.Phil/MS/PhD
Other	

9. **How many years of education or training have you completed?**

Total years of education or training: ✐.................. *(Number of years)*

Years of education or training acquired after your spinal cord injury: ✐.................. *(Number of years)*

10 **Taking into account all persons living in your household who work for a salary or wage: what is the total household income after taxes on average per month?**

Decile 1 (a)	BDT 0-10,200
Decile 1 (b)	BDT 10,201- 29,000
Decile 1 (c)	BDT 29,001- 34,682
Decile 2(a)	BDT 34,683- 50,000
Decile 2(b)	BDT 50,001- 102312
Decile 3	102313-126545
Decile 4	126546- 152278
Decile 5	152279- 182041
Decile 6	182042-219924
Decile 7	219925-273087
Decile 8	273088-349848
Decile 9	349849-517922
Decile 10	517923-above

11 **How satisfied are you with the financial situation of your household?**

Completely dissatisfied ← → *Completely satisfied*

○	○	○	○	○	○	○	○	○	○
1	2	3	4	5	6	7	8	9	10

12 **Think of this ladder as representing where people stand in *[country]*.**

At the top of the ladder are the people who are the best off - those who have the most money, the most education and the most respected jobs. At the bottom are the people who are the worst off – who have the least money, least education, and the least respected jobs or no job. The higher up you are on this ladder, the closer you are to the people at the very top; the lower you are, the closer you are to the people at the very bottom.

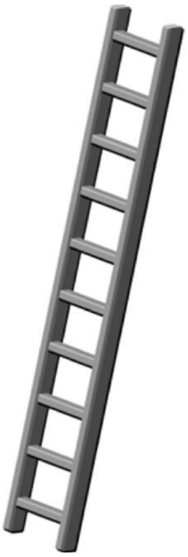

Where would you place yourself on this ladder?

Please place a large **X** on the rung where you would place yourself at this time in your life, relative to other people in **[your country]**

Lesion characteristics

13 Please describe the level of your spinal cord injury:

- ○ Paraplegia (affects lower limbs with normal movement and feeling in the upper limbs)
- ○ Tetraplegia (absent or abnormal movement or feeling in the upper and lower limbs)
- ○ Fully recovered (normal feeling and ability to move, no symptoms of bladder or bowel impairment due to SCI)

14 Is your injury complete or incomplete?

- ○ Complete (unable to feel and move any part of your body below injury level)
- ○ Incomplete (able to feel or move some part/s of your body below injury level)
- ○ Fully recovered (normal feeling and ability to move, no symptoms of bladder or bowel impairment due to SCI)

15 Please indicate the cause of your spinal cord injury:

Caused by injury:

Check all that apply

For example, if you check the box 'accident during work', please also specify if it was a fall or another cause of injury.

- ☐ Accident during sports
- ☐ Accident during leisure activity
- ☐ Accident during work
- ☐ Traffic accident (RTA)
- ☐ Injury due to violence *e.g., gunshot wound*
- ☐ Strangulation suicidal ligature
- ☐ Strangulation accidental (e.g. scarf injury/ligature)
- ☐ Strangulation homicidal
- ☐ Fall from less than 1 meter
- ☐ Fall from more than 1 meter
- ☐ Consequence of a medical intervention or surgery
- ☐ Other cause of injury: ✎...

***Caused by disease*:**

Check all that apply

- ☐ Degeneration of the spinal column
- ☐ Tumor – benign
- ☐ Tumor – malignant (cancer)
- ☐ Vascular problem *e.g., ischemia, hemorrhage, malformations*
- ☐ Infection *e.g., bacterial, viral*
- ☐ Other disease: ✎...

**16**	**Please indicate as precisely as possible the date on which your spinal cord injury occurred:**<br><br>DD / MM / YYYY<br>□□/□□/□□□□
**16.1**	**Have you undergone any surgery due to spinal cord injury?**<br><br>○ Yes<br>○ No

**16.2**	**If yes, within which time period?**<br><br>○ Within 3 days<br>○ Between 3-7days<br>○ After 7 days
**16.3**	**Who is your main contact for spinal cord injury specific problems?**<br>○ General practitioner<br>○ Spinal specialist working in a specialist spinal cord injury service/unit<br>○ Physical Medicine Rehabilitation specialist<br>○ Other local specialist (e.g. urologist, neurologist)<br>○ Others, namely:
**16.4**	**Have you visited any Physical Medicine Rehabilitation doctor/rehabilitation hospital for this reason?**<br><br>○ Yes<br>○ No
**16.5**	**How satisfied are you with the services provided by the Spinal Cord Injury Unit/Service/s in your district?**<br>○ Very satisfied<br>○ Satisfied<br>○ Neither satisfied nor dissatisfied<br>○ Dissatisfied<br>○ Very dissatisfied<br>○ I don't use this service<br>○ I don't know about the service

Energy and feelings

These questions are about how you have felt and how things have been with you during the last 4 weeks. For each question, please give the one answer that comes closest to the way you have been feeling.

**17 How much of the time during the last 4 weeks…**		*All of the time*	*Most of the time*	*Some of the time*	*A little of the time*	*None of the time*
**a.**	**Did you feel full of life?**	○	○	○	○	○
**b.**	**Have you been very nervous?**	○	○	○	○	○
**c.**	**Have you felt so down in the dumps that nothing could cheer you up?**	○	○	○	○	○
**d.**	**Have you felt calm and peaceful?**	○	○	○	○	○
**e.**	**Did you have a lot of energy?**	○	○	○	○	○
**f.**	**Have you felt downhearted and depressed?**	○	○	○	○	○
**g.**	**Did you feel worn out?**	○	○	○	○	○
**h.**	**Have you been happy?**	○	○	○	○	○
**i.**	**Did you feel tired?**	○	○	○	○	○
		1 No Problem	*2*	*3*	*4*	*5 Extreme problem*

18	**How much of a problem is feeling tired or not having enough energy?**	O	O	O	O	O

19 For the following health problems please rate how much of a problem it was for you in the last 3 months. If you have experienced the health problem, please indicate whether you have received treatment or not (e.g., taking a medication or getting treatment by doctors or other health professionals).

		1 No Problem	*2*	*3*	*4*	*5 Extreme problem*	*Do/did you receive treatment for it?*
a.	**Sleep problems** *e.g., problems falling asleep or sleeping through the night and waking up early.*	O	O	O	O	O	O Yes<br>O No
b.	**Bowel dysfunction** *e.g., diarrhea, stool incontinence ('accidents') and constipation.*	O	O	O	O	O	O Yes<br>O No
c.	**Urinary tract infections** *e.g., kidney or bladder infection.*	O	O	O	O	O	O Yes<br>O No
d.	**Bladder dysfunction** *e.g., incontinence ('accidents'), bladder or kidney stones, kidney problems, urine leakage and urine back up.*	O	O	O	O	O	O Yes<br>O No
e.	**Sexual dysfunction** *e.g., difficulty with sexual arousal, erection, lubrication, and reaching orgasm.*	O	O	O	O	O	O Yes<br>O No
f.	**Contractures** *This is a limitation in the range of motion of a joint.*	O	O	O	O	O	O Yes<br>O No
g.	**Muscle spasms, spasticity** *This refers to uncontrolled, jerky muscle movements, such as uncontrolled muscle twitches or spasms.*	O	O	O	O	O	O Yes<br>O No
h.	**Pressure sores, decubitus** *These develop as a skin rash or redness and may progress to an infected sore.*	O	O	O	O	O	O Yes<br>O No
i.	**Respiratory problems** *Symptoms of respiratory infections or problems include difficulty in breathing and increased secretions.*	O	O	O	O	O	O Yes<br>O No

		1<br>No<br>Problem	2	3	4	5<br>Extreme problem	Do/did you receive treatment for it?
j.	**Injury caused by loss of sensation**<br>*e.g., burns from carrying hot liquids in the lap or sitting too close to a heater or fire.*	○	○	○	○	○	○ Yes<br>○ No
k.	**Circulatory problems**<br>*This involves the swelling of veins, feet, legs or hands, or the occurrence of blood clots.*	○	○	○	○	○	○ Yes<br>○ No
l.	**Autonomic dysreflexia**<br>*Symptoms are sudden rises in blood pressure and sweating, skin blotches, goose bumps, pupil dilation and headache.*	○	○	○	○	○	○ Yes<br>○ No
m.	**Postural hypotension**<br>*This involves a strong sensation of lightheadedness following a change in position. It is caused by a sudden drop in blood pressure.*	○	○	○	○	○	○ Yes<br>○ No
n.	**Pain**<br>*Having pain in your day-to-day life.*	○	○	○	○	○	○ Yes<br>○ No
**19.1**	Have you had any pain during the last seven days including today?<br>No (go to Question 21)<br>**Yes** If yes, please answer the following questions about the extent to which pain interferes with your life and how it is being managed:						
**20**	**Please rate your pain by circling the number that best describes your pain at its worst in the last week.**						

No pain										*Pain as bad as you can imagine*
○	○	○	○	○	○	○	○	○	○	○
0	1	2	3	4	5	6	7	8	9	10

20.1	How would you describe your pain?	☐ I experience pain that is hot or burning, cold or freezing, pins and needles, tingling, electric shock-like or similar in quality.<br>☐ I experience pain that is dull, aching, cramping or tender in muscles in an area or normal sensation.<br>☐ The pain only occurs in an area of the body in which I have no feeling on the skin overlying that area.<br>☐ The skin over the area of pain is abnormally sensitive to touch and without any surgical scars, ulcers or breaks in the skin.<br>☐ The pain is usually unchanged with movement of the painful area.<br>☐ The pain is made worse by certain movements, postures or activities.

		☐ I experience pain all the time without any breaks when I am awake (although it may vary in intensity during different times).

20.2 **In general, how much has pain interfered with your day-to-day activities <u>in the last week</u>?**

No Interference ← → *Extreme interference*

☐	☐	☐	☐	☐	☐	☐	☐	☐	☐	☐
0	1	2	3	4	5	6	7	8	9	10

20.3 **In general, how much has pain interfered with your overall mood <u>in the last week</u>?**

No Interference ← → *Extreme interference*

☐	☐	☐	☐	☐	☐	☐	☐	☐	☐	☐
0	1	2	3	4	5	6	7	8	9	10

20.4 **In general, how much has pain interfered with your ability to get a good night's sleep in the last week?**

No Interference ← → *Extreme interference*

☐	☐	☐	☐	☐	☐	☐	☐	☐	☐	☐
0	1	2	3	4	5	6	7	8	9	10

20.5 **Which of these treatments/strategies do you use to help manage your pain – *select all that apply*?**

Check all that apply

- Take over the counter medicines (e.g. Paracetamol, Ibuprofen)
- Take prescribed nerve pain medications (e.g. Lyrica, Neurontin)
- Take prescribed opioid medications (e.g. Endone, Oxycodone, Fentanyl patch)
- Take non-prescribed medications (e.g. marijuana)
- Use alcohol to dull the pain
- Attend physiotherapy
- Seek emotional or psychological support
- Attend peer support groups
- Keep physically active (e.g. walking, fitness programs, daily chores)
- Try to get enough sleep
- Practice relaxation / meditation (e.g. mindfulness) techniques regularly
- Try to eat a healthy diet and keep to a healthy weight
- Attend manual therapies (e.g. massage, acupuncture, osteopathy, chiropractic)
- Receive other complementary therapies or treatments (e.g. homeopathy, naturopathy, Chinese herbalism)
- Others, please specify ☐...

20.6 **Please rate how effective you find the treatments you use for managing your pain?**

Ineffective ← → *Extremely effective*

☐	☐	☐	☐	☐	☐	☐	☐	☐	☐	☐
0	1	2	3	4	5	6	7	8	9	10

20.7 **Where do you receive advice and help to manage your pain – *select all that apply*?**

Check all that apply

☐ General Practitioner
☐ Spinal Specialist
☐ Pain Specialist

		☐ Physical therapist (eg: physiotherapist, exercise physiologist, occupational therapist)<br>☐ Psychologist / Counsellor<br>☐ Hospital (including Emergency Department)<br>☐ Chronic Pain Clinic (which provides multi-disciplinary care)<br>☐ Online Pain Management Courses (e.g. e-centre clinic)<br>☐ Complementary therapist (e.g. acupuncturist, homeopath, naturopath, Chinese herbalist)<br>☐ Website (e.g. ACI Pain Management Network - SCI Pain pages)<br>☐ Other, please specify: ……………

**21**	**Please name additional health problems (co-morbidities) that also bother you. Do not repeat from the health problems mentioned in the section before.**<br>☐ No additional health problem experienced<br><br>*Choose the most common problems (multiple options)*<br>☐ Heart disease<br>☐ Diabetes<br>☐ Cancer<br>☐ Hypertension<br>☐ Other ✎……………………
**21.1**	**Blood Pressure Measurement**<br>○ ……mmHg (systolic)<br>○ ……mmHg (Diastolic)

**22**	**Please indicate your current smoking status:**<br>○ Never smoked<br>○ Former smoker<br>○ Current smoker (including occasional smoker)
**22.1**	**Are you a smokeless tobacco user?**<br>○ Yes<br>○ No
**22.2**	**Have you use any type of alcohol or drug?**<br>○ Yes<br>○ No
**23**	**Please indicate your approximate height and weight:**

	✐...................... in kg. (weight)<br><br>✐...................... in cm. (height)

Activity and participation

The following section is about problems you experience in your life. Please take both good and bad days into account.

24.	In the last 4 weeks, how much of a problem have you had...	*1 No problem*	*2*	*3*	*4*	*5 Extreme problem*
a.	... carrying out daily routine?	○	○	○	○	○
b.	... handling stress?	○	○	○	○	○
c.	... doing things that require the use of your hands and fingers, such as picking up small objects or opening a container?	○	○	○	○	○
d.	... getting where you want to go?	○	○	○	○	○
e.	... using public transportation?	○	○	○	○	○
f.	... using private transportation?	○	○	○	○	○
g.	... looking after your health, eating well, exercising or taking your medicine?	○	○	○	○	○
h.	... getting your household tasks done?	○	○	○	○	○
i.	... providing care or support for others?	○	○	○	○	○
j.	... interacting with people?	○	○	○	○	○
k.	... with intimate relationships?	○	○	○	○	○
l.	... doing things for relaxation or pleasure?	○	○	○	○	○
m.	... with shortness of breath during physical exertion?	○	○	○	○	○
	**In the last 4 weeks, how much of a problem have you had...**	*1 No problem*	*2*	*3*	*4*	*5 Extreme problem*
25a.	... to sit without support for long periods such as 30 minutes?	○	○	○	○	○
25b.	... to stand without support for long periods such as 30 minutes?	○	○	○	○	○

These questions ask about your ability to do activities that involve mobility. Select the response that best describes your ability to do the activity without help from another person but using the equipment or devices you normally use (e.g., transfer boards lifts, hospital bed).

26.	Are you able to…	*Without any difficulty*	*With a little difficulty*	*With some difficulty*	*With much difficulty*	*Unable to do*
a.	… get up off the floor from lying on your back?	○	○	○	○	○
b.	… push open a heavy door?	○	○	○	○	○
c.	… moving from sitting at the side of the bed to lying down on your back?	○	○	○	○	○

Independence in activities of daily living

For each item, please check the box next to the statement that best reflects your current situation. Please read the text carefully and only check one box in each section.

Self Care

27. Eating and drinking

- ○ I need artificial feeding or a stomach tube
- ○ I need total assistance with eating / drinking
- ○ I need partial assistance with eating / drinking or for putting on/taking off adaptive devices
- ○ I eat / drink independently, but I need adaptive devices or assistance for cutting food, pouring drinks or opening containers
- ○ I eat / drink independently without assistance or adaptive devices

28. Washing your upper body and head

This includes soaping and drying, and using a water tap.

- ○ I need total assistance
- ○ I need partial assistance
- ○ I am independent but need adaptive devices or specific equipment *e.g., bars, chair*
- ○ I am independent and do not need adaptive devices or specific equipment

29. Washing your lower body

This includes soaping and drying, and using a water tap.

- ○ I need total assistance
- ○ I need partial assistance
- ○ I am independent but need adaptive devices or specific equipment *e.g., bars, chai*
- ○ I am independent and do not need adaptive devices or specific equipment

30. Dressing your upper body

This includes putting on and taking off clothes like t-shirts, blouses, shirts, bras, shawls, or orthoses (e.g., arm splint, neck brace, corset).

- *Easy-to-dress clothes are those without buttons, zippers or laces*
- *Difficult-to-dress clothes are those with buttons, zippers or laces*

- ○ I need total assistance
- ○ I need partial assistance, even with easy-to-dress clothes
- ○ I do not need assistance with easy-to-dress clothes, but I need adaptive devices or specific equipment
- ○ I am independent with easy-to-dress clothes and only need assistance or adaptive devices or a specific setting with difficult-to-dress clothes

- ○ I am completely independent

31. Dressing your lower body

This includes putting on and taking off clothes like shorts, trousers, shoes, socks, belts, or orthoses (e.g., leg splint).

- *Easy-to-dress clothes are those without buttons, zippers or laces*
- *Difficult-to-dress clothes are those with buttons, zippers or laces*

- ○ I need total assistance
- ○ I need partial assistance, even with easy-to-dress clothes
- ○ I do not need assistance with easy-to-dress clothes, but I need adaptive devices or specific equipment
- ○ I am independent with easy-to-dress clothes and only need assistance or adaptive devices or a specific setting with difficult-to-dress clothes
- ○ I am completely independent

32. Grooming

e.g., activities such as washing hands and face, brushing teeth, combing hair, shaving, or applying make-up.

- ○ I need total assistance
- ○ I need partial assistance
- ○ I am independent with adaptive devices
- ○ I am independent without adaptive devices

Breathing

33. I need a respiratory (tracheal) tube …

- ○ and permanent or assisted ventilation from time to time.
- ○ and extra oxygen and much assistance in coughing or respiratory (tracheal) tube management.
- ○ and little assistance in coughing or respiration (tracheal) tube management.

I do not need a respiratory (tracheal) tube…

- ○ but I need extra oxygen or much assistance in coughing or a mask (e.g. PEEP) or assisted ventilation from time to time (e.g. BIPAP).
- ○ and only little assistance or stimulation for coughing.
- ○ and can breathe and cough independently without assistance or device.

Bladder and bowel management

34. Bladder management

Please think about the way you empty your bladder.

A. Use of an indwelling catheter

- ○ Yes → *Please go to question no. 35*
- ○ No → *Please also answer B and C.*

B. Intermittent catheterization

- ○ I need total assistance
- ○ I do it myself with assistance (self-catheterization)
- ○ I do it myself without assistance (self-catheterization)
- ○ I do not use it

***C.* Use of external drainage instruments** *e.g., condom catheter, diapers, sanitary napkins*

- ○ I need total assistance for using them
- ○ I need partial assistance for using them
- ○ I use them without assistance

○ I am continent with urine and do not use external drainage instruments

35. Bowel management

A. Do you need assistance with bowel management *e.g., for applying suppositories*?

○ Yes
○ No

B. My bowel movements are…

○ irregular or seldom (less than once in 3 days)
○ regular (once in 3 days or more)

C. Fecal incontinence ("accidents") happens …

○ Daily
○ 1-6 times per week
○ 1-4 times every month
○ Less than once per month
○ Never

36. Using the toilet

Please think about the use of the toilet, cleaning your genital area and hands, putting on and taking off clothes, and the use of sanitary napkins or diapers.

○ I need total assistance
○ I need partial assistance and cannot clean myself
○ I need partial assistance but can clean myself
○ I do not need assistance but I need adaptive devices (*e.g., bars)* or a special setting *(e.g., wheelchair accessible toilet)*
○ I do not need any assistance, adaptive devices or a special setting

Mobility

37. Which of the following activities can you perform without assistance or electrical aids?

Check all that apply

☐ Turning your upper body in bed
☐ Turning your lower body in bed
☐ Sitting up in bed
☐ Doing push-ups in in a chair or wheelchair
☐ None, I need assistance in all these activities

38. Transfers from the bed to the wheelchair

○ I need total assistance
○ I need partial assistance, supervision or adaptive devices e.g., *sliding board*
○ I do not need any assistance or adaptive devices
○ I do not use a wheelchair

39. Moving around moderate distances (10 to 100 meters)

I use a wheelchair. To move around, …

○ I need total assistance
○ I need an electric wheelchair or partial assistance to operate a manual wheelchair
○ I am independent in a manual wheelchair

I walk moderate distances and I ...

○ need supervision while walking (with or without walking aids)
○ walk with a walking frame or crutches, swinging forward with both feet at a time
○ walk with crutches or two canes, setting one foot before the other

○ walk with one cane
○ walk with a leg orthosis(es) only *e.g., leg splint*
○ walk without walking aids

Work

40. **What was the name or title of your main job before your spinal cord injury?**

○ I did not have a job before my spinal cord injury.
○ The name or title of my main job was as follows:

✐..

Please be as specific as possible, e.g., not just 'clerk' but 'bank clerk'; not just 'manager' but 'sales manager'

40.1

	Very satisfied	*Rather satisfied*	*Partly satisfied/partly dissatisfied*	*Rather dissatisfied*	*Very dissatisfied*
**How satisfied were you with your job before the onset of your spinal cord injury?**					

41. **Did you receive vocational rehabilitation services after your spinal cord injury?**
e.g., vocational counseling, vocational retraining, job skills training

○ Yes
○ No

42. **After your discharge from initial inpatient rehabilitation, how long did it take before you started or resumed paid work?**

○ I never worked after initial inpatient rehabilitation
○ Immediately after initial rehabilitation
○ I resumed work after ✐.............. years and ✐.............. months

43. **Do you currently receive a disability pension or a similar disability benefit?**

○ Yes
○ No

44. **What is your current working situation?**
Check all that apply

☐ Working for wages or salary with an employer for ✐.............. hours a week
☐ Working for wages with an employer for ✐.............. hours a week, but currently on sick leave for more than three months
☐ Self-employed, working for ✐.............. hours a week
☐ Working as unpaid family member *e.g., working in family business*
☐ Housewife / househusband
☐ Student
☐ Unemployed
☐ Retired due to the health condition
☐ Retired due to age
☐ Other, please specify: ✐..

45. **Are you currently engaged in paid work?**

	○ Yes<br>○ No → *Please go to question no. 50*
**46.**	**What is the name or title of your current main job?**<br>*Please be as specific as possible, e.g., not just 'clerk' but 'bank clerk'; not just 'manager' but 'sales manager'*<br>✎ ....

46.1		*Very satisfied*	*Rather satisfied*	*Partly satisfied/partly dissatisfied*	*Rather dissatisfied*	*Very dissatisfied*
	**How satisfied are you with your job today?**					

47. Do you want to work more, less or the same number of hours as you currently do?

○ More hours
○ Less hours
○ The same amount

48.		*Completely*	*To a large extent*	*To some extent*	*To a small extent*	*Not at all*	*I do not have such a need*
	**Do you have the assistive devices that you need for work?**<br>*e.g., assistive computer devices, adjustable desks or arm/hand braces or prosthetics*	○	○	○	○	○	○

The following two questions refer to your present occupation. For each of the following statements, please indicate whether you strongly agree, agree, disagree or strongly disagree.

49		*Strongly agree*	*Agree*	*Disagree*	*Strongly disagree*
a	**I receive the recognition I deserve for my work.**	○	○	○	○
b	**Considering all my efforts and achievements, my salary is adequate.**	○	○	○	○

49.1	**Please indicate to what extent you agree with the following statements on your current job.**	*Strongly agree*	*Agree*	*Disagree*	*Strongly disagree*
a.	**I am under constant time pressure due to a heavy work load**	○	○	○	○
b.	**I have many interruptions and disturbances while performing my job**	○	○	○	○

c.	Over the past few years, my job has become more and more demanding	○	○	○	○
d.	I receive the respect I deserve from my superior or a respective relevant person	○	○	○	○
e.	My job promotion prospects are poor	○	○	○	○
f.	I have experienced or I expect to experience an undesirable change in my work situation	○	○	○	○
g.	My job security is poor	○	○	○	○
h.	Considering all my efforts and achievements, I receive the respect and prestige I deserve at work	○	○	○	○
i.	Considering all my efforts and achievements, my job promotion prospects are adequate	○	○	○	○

The following three questions refer to your present occupation. How much does the management at your workplace allow you to do the following on a scale from 0 no influence) to 10 (complete control) …?

49.2
…to decide how your own daily work is organized?

no influence ← → *Complete control*

○	○	○	○	○	○	○	○	○	○	○
0	1	2	3	4	5	6	7	8	9	10

49.3
...to choose or change your pace of work?

no influence ← → *Complete control*

○	○	○	○	○	○	○	○	○	○	○
0	1	2	3	4	5	6	7	8	9	10

49.4
…to influence policy decisions about the activities of the organization?

no influence — *Complete control*

0	1	2	3	4	5	6	7	8	9	10
○	○	○	○	○	○	○	○	○	○	○

→ *Please go to question no. 52.1*

50. Would you like to have paid work?

- ○ Yes
- ○ No

51. Do you feel able to perform paid work?

- ○ Yes, for 1 – 11 hours a week
- ○ Yes, for 12 – 20 hours a week
- ○ Yes, for more than 20 hours a week
- ○ No, not at all

52. What are the reasons you are not currently working?

Check all that apply

- ☐ Health condition or disability
- ☐ Still engaged in educational or vocational training
- ☐ Personal family responsibilities
- ☐ Could not find suitable work
- ☐ Do not know how or where to seek work
- ☐ Do not have the financial need
- ☐ Parents or spouse did not let me work
- ☐ Insufficient transportation services
- ☐ Lack of accessibility to potential workplaces *e.g., access to the building, your office or toilets*
- ☐ Lack of assistive devices
- ☐ Fear of losing disability benefits *e.g., pension payments, health insurance coverage*
- ☐ I do not want to work
- ☐ Other, please specify: ✎ ……………………………………………………………………………..

52.1 Do you receive any social benefits from any source?

- ○ No (please go to 52.5)
- ○ Yes

52.2 If yes, Then from which source?

- ○ Government (please go to 52.4)
- ○ Private
- ○ NGO
- ○ Other sources, please specify.

52.3 How satisfied are you social benefits given by the Government?

- ○ Very satisfied
- ○ Rather satisfied
- ○ Partly satisfied/partly dissatisfied
- ○ Rather dissatisfied

○ Very dissatisfied

52.4 How *easy it is to get social benefits given by the Government*? (0 very easy and 10 very difficult)

Very Easy *Very difficult*

0	1	2	3	4	5	6	7	8	9	10
▯	▯	▯	▯	▯	▯	▯	▯	▯	▯	▯

52.5 How *easy it is to get employment for person with SCI* in *Bangladesh*?

OR How *easy it is to continue education for person with SCI in Bangladesh*? (0 very easy and 10 very difficult)

Very Easy *Very difficult*

0	1	2	3	4	5	6	7	8	9	10
▯	▯	▯	▯	▯	▯	▯	▯	▯	▯	▯

52.6 Recommendations

Do you have any comments/wishes/suggestions how to improve the labour market integration of people with an SCI?

………

Environmental factors

53. In daily life, we are exposed to various external influences or environmental factors. These can make daily life easier or more difficult. Thinking about the last 4 weeks, please rate how much these environmental factors have influenced your participation in society.

		Not applicable	*No influence*	*Made my life a little harder*	*Made my life a lot harder*
a.	**Missing or insufficient accessibility of public places**<br>*e.g., inaccessible public buildings, parks*	○	○	○	○
b.	**Missing or insufficient accessibility to the homes of friends and relatives**	○	○	○	○
c.	**Unfavorable climatic conditions**<br>*e.g., weather, season, temperature, humidity*	○	○	○	○
d.	**Negative societal attitudes toward persons with disability**<br>*e.g., prejudice, stigma, ignorance*	○	○	○	○
e.	**Negative attitudes of your family and relatives with regards to your disability**<br>*e.g., prejudice, lack of support, overprotective behavior*	○	○	○	○
f.	**Negative attitudes of your friends with regards to your disability**<br>*e.g., prejudice, lack of support, overprotective behavior*	○	○	○	○
g.	**Negative attitudes of neighbors, acquaintances and work colleagues with regards to your disability**<br>*e.g., prejudice, lack of support, overprotective behavior*	○	○	○	○
h.	**Lack of or insufficient adapted assistive technology for moving around over short distances**<br>*e.g., stair lift, walking aids or wheelchair*	○	○	○	○

i.	**Lack of or inadequate adapted means of transportation for long distances** *e.g., lack of adapted car or hard to use public transportation*	○	○	○	○
j.	**Lack of or insufficient nursing care and support services** *e.g., home health care or personal assistance*	○	○	○	○
k.	**Lack of or insufficient medication and medical aids and supplies** *e.g., catheters, disinfectants, splints, pillows*	○	○	○	○
l.	**Problematic financial situation** *e.g., shortage of money*	○	○	○	○
m.	**Lack of or insufficient communication devices** *e.g., lack of or insufficient writing devices, computer, telephone, mouse*	○	○	○	○
n.	**Lack of or insufficient state services** *e.g., disability insurance or other benefits*	○	○	○	○

Health care services

54. Who were the health care providers you visited, or who visited you in your home, in the last 12 months?

- ☐ I did not visit any health care provider in the last 12 months.

Check all that apply

- ☐ Primary care physician / general practitioner
- ☐ Physical Medicine Rehabilitation physician / spinal cord injury physician
- ☐ Other specialist physician *e.g., surgeon/, gynecologist, /psychiatrist,/ ophthalmologist*
- ☐ Nurse
- ☐ Psychologist
- ☐ Physiotherapist
- ☐ Chiropractor
- ☐ Occupational therapist
- ☐ Others, please specify: ✎ ....

55. Over the last 12 months, how many times were you a patient in a hospital, rehabilitation facility or another care facility for at least one night?

✎ .... *(times)*

56.	**For your last visit to a GP or to a primary healthcare provider, how would you rate the following:**	*Very satisfied*	*Rather satisfied*	*Neither satisfied nor dissatisfied*	*Rather dissatisfied*	*Very dissatisfied*	*I do not use the service*
a.	**...your experience of being treated respectfully?**	○	○	○	○	○	○
b.	**...how clearly health care providers explained things to you?**	○	○	○	○	○	○
c.	**...your experience of being involved in making decisions for your treatment?**	○	○	○	○	○	○

57. In the last 12 months, have you needed health care but did not get it?

- ○ No
- ○ Yes. Which reason(s) explain why you did not get the health care you needed?

Check all that apply

- ☐ Could not afford the cost of the visit
- ☐ There was no service
- ☐ No transport available
- ☐ Could not afford the cost of transportation
- ☐ You were previously badly treated
- ☐ Could not take time off work or had other commitments
- ☐ The health care provider's drugs or equipment were inadequate
- ☐ The health care provider's skills were inadequate
- ☐ You did not know where to go
- ☐ You tried but were denied health care
- ☐ You thought you were not sick enough
- ☐ The service was temporarily not available due to the Covid-19 pandemic
- ☐ Other, please specify: ✎ ....

	Very satisfied	*Satisfied*	*Neither satisfied nor dissatisfied*	*Dissatisfied*	*Very dissatisfied*
**58. In general, how satisfied are you with how the health care services are run in your area?**	○	○	○	○	○

Covid-19 and Vaccinations

59. Have you ever been diagnosed with COVID-19 (a positive laboratory test or confirmed by a healthcare provider)?

- ○ No → *Please go to question no.61*
- ○ I don't know → *Please go to question no.61*
- ○ Yes

60. How long did your symptoms last?

✎................. (months) .... (weeks)

- ○ I still suffer from symptoms (e.g., muscle and joint pain, weakness, or fatigue)

61. Since the beginning of the pandemic, have you been hospitalized due to COVID-19 symptoms?

- ○ Yes
- ○ No

62. Have you been vaccinated for COVID-19?

- ○ Yes
- ○ No, I do not want to
- ○ No, I could not get vaccinated
- ○ No, I had no access

Personal factors

The following questions are about how you see yourself.

63.		1<br>Not at all	2	3	4	5<br>Completely
a.	How confident are you that you can find the means and ways to get what you want if someone opposes you?	○	○	○	○	○
b.	How confident are you that you could deal efficiently with unexpected events?	○	○	○	○	○
c.	How confident are you that you can maintain contact with people who are important to you?	○	○	○	○	○
d.	How confident are you that you can maintain good health?	○	○	○	○	○
e.	How confident are you that you can maintain your personal hygiene with or without help?	○	○	○	○	○
f.	How confident are you that you can find hobbies or leisure activities that interest you?	○	○	○	○	○
g.	Do you think that living with your spinal cord injury has made you a stronger person?	○	○	○	○	○
h.	Do you feel that you will be able to achieve your dreams, hopes, and wishes?	○	○	○	○	○
i.	Do you feel included when you are with other people?	○	○	○	○	○
j.	I have a good sense of what makes my life meaningful	○	○	○	○	○
k.	I am searching for meaning in my life	○	○	○	○	○

		Rarely true	*Sometim es true*	*Often true*	*True nearly*	*All the time*
**64.**	**I feel completely alone**	○	○	○	○	○

Quality of life and general health

The next questions are about how you rate your quality of life over the last 14 days. Please keep in mind your standards, hopes, pleasures and concerns.

	**In the last 14 days…**	*Very poor*	*Poor*	*Neither poor nor good*	*Good*	*Very good*
**65.**	**How would you rate your quality of life?**	○	○	○	○	○

66.		Very dissatisfied	Dissatisfied	Neither satisfied nor dissatisfied	Satisfied	Very satisfied
a.	**How satisfied are you with your health?**	○	○	○	○	○
b.	**How satisfied are you with your ability to perform your daily living activities?**	○	○	○	○	○
c.	**How satisfied are you with yourself?**	○	○	○	○	○
d.	**How satisfied are you with your personal relationships?**	○	○	○	○	○
e.	**How satisfied are you with your living conditions?**	○	○	○	○	○

67. In general, would you say your health is:

- ○ Excellent
- ○ Very good
- ○ Good
- ○ Fair
- ○ Poor

68. Compared to one year ago, how would you rate your health in general now?

- ○ Much better
- ○ Somewhat better
- ○ About the same
- ○ Somewhat worse
- ○ Much worse

Physical Activity

This section is about your current level of physical activity and exercise. There are no right or wrong answers, we simply need to assess your current level of activity.

These questions measure the number of minutes of light, moderate and heavy intensity and strength physical activity performed over the previous 7 days.

Please recall the physical activity you have completed **over the previous 7 days only**. Each question requests you to recall how many days (out of the previous 7) you performed the type of physical activity, and for how many minutes you usually performed that type of physical activity each day.

Exercise Information:

Leisure-Time Physical Activity: activities that you choose to do during your free time, such as exercising, playing sports, swimming, gardening or taking the dog for a walk. Necessary physical activities that you have to do such as physiotherapy, grocery shopping and pushing/wheeling for transportation are not considered leisure-time physical activities.

Aerobic activities: activities that are done continuously and that increase your heart rate and breathing rate, such as walking, wheeling, swimming, hand cycling or dancing

Strength-training activities: activities that increase muscle strength, such as exercises using resistance bands or lifting weights.

Exercise Intensity:

Moderate intensity aerobic physical activity requires some physical effort. Moderate intensity activities make you feel like you are working somewhat hard, but you can keep doing them for a while without getting tired.

Vigorous intensity aerobic physical activity requires a lot of physical effort. Heavy intensity activities make you feel like you are working really hard, almost at your maximum. You cannot do these activities for very long without getting tired. These activities may be exhausting.

For each intensity, please input the number of days and the number of minutes that you performed ***aerobic activities in the last seven days***

**69.**	**What is the total duration per week you perform moderate to vigorous aerobic activities?**<br>*Physical activities that are done continuously and that increase your heart rate and breathing rate, such as wheeling (for leisure), swimming, hand cycling or dancing.*<br>○ Never<br>○ < 40 minutes<br>○ 40-59 minutes<br>○ 60-90 minutes<br>○ > 90 minutes
**69.1**	**What are the total number of days per week you perform moderate to vigorous aerobic activities?**<br>**….. days**
**70.**	**Do you do muscle specific strength training?**<br>*Strength-training activities are activities that increase muscle strength, such as exercises using resistance bands, or lifting weights, 3 sets of 8-10 repetitions of each exercise for each major muscle.*<br>○ No<br>○ Once a week<br>○ twice a week<br>○ more than twice a week
**70.1**	**Duration of muscle specific strength training?**<br>○ Never<br>○ < 40 minutes<br>○ 40-59 minutes<br>○ 60-90 minutes<br>○ > 90 minutes

Health Utility

Under each heading, please tick the ONE box that best describes your health TODAY.

71. **Mobility**

- ○ I have no problems in walking about
- ○ I have slight problems in walking about
- ○ I have moderate problems in walking about
- ○ I have severe problems in walking about
- ○ I am unable to walk about

72. **Self-care**

- ○ I have no problems washing or dressing myself
- ○ I have slight problems washing or dressing myself
- ○ I have moderate problems washing or dressing myself
- ○ I have severe problems washing or dressing myself
- ○ I am unable to wash or dress myself

73. **Usual activities *(e.g. work, study, housework, family or leisure activities)***

- ○ I have no problems doing my usual activities
- ○ I have slight problems doing my usual activities
- ○ I have moderate problems doing my usual activities
- ○ I have severe problems doing my usual activities
- ○ I am unable to do my usual activities

74. **Pain / Discomfort**

- ○ I have no pain or discomfort
- ○ I have slight pain or discomfort
- ○ I have moderate pain or discomfort
- ○ I have severe pain or discomfort
- ○ I have extreme pain or discomfort

75. **Anxiety /Depression**

- ○ I am not anxious or depressed
- ○ I am slightly anxious or depressed
- ○ I am moderately anxious or depressed
- ○ I am severely anxious or depressed
- ○ I am extremely anxious or depressed

76. **The next four questions ask about the impact of fatigue on your functioning. Please choose a number for each statement from 1 to 7 that indicates your level of agreement with each statement, where 1 indicates "Strongly disagree" and 7 indicates "Strongly agree"**

		1<br>*Strongly disagree*	*2*<br>*Disagree*	*3*<br>*Slightly disagree*	*4*<br>*Neither agree nor disagree*	*5*<br>*Slightly agree*	*6*<br>*Agree*	*7*<br>*Strongly agree*
**a.**	**I am easily fatigued.**							
**b.**	**Fatigue interferes with my physical functioning.**							
**c.**	**Fatigue causes me frequent problems.**							

d.	**Fatigue interferes with my work, family or social life.**							
e.	**Exercise brings on my fatigue**							

77. **What is your current living arrangement?**

- ○ Private residence – Owned (with mortgage or outright)
- ○ Private residence – Rented from a Real estate agent or private landlord
- ○ Private residence – Rented from a Government Housing Department (Public Housing) or a Community housing provider
- ○ Private residence – Rented from Parent or other relative not in this dwelling; or occupied rent-free.
- ○ A dwelling occupied with a 'life tenure' scheme (e.g. in a retirement village or similar arrangement)
- ○ Residential Aged Care Facility / Nursing Home
- ○ Interim / Transitional / Short- or Medium-term accommodation
- ○ No Home / accommodation
- ○ Others, please specify ☐...……….
- ○ Cluster house

78. **To what extent do you feel that you have the information you need to live with and manage your condition and to make decisions?**

Not at all ⟵⟶ *Completely*

☐	☐	☐	☐	☐	☐	☐	☐	☐	☐	☐
0	1	2	3	4	5	6	7	8	9	10

79. **To what extent do you feel able to find out about services and supports in order to meet your needs?**

Not at all ⟵⟶ *Completely*

☐	☐	☐	☐	☐	☐	☐	☐	☐	☐	☐
0	1	2	3	4	5	6	7	8	9	10

80. **Have you used telehealth/telemedicine services in the last 12 months**

(Telehealth is the provision of healthcare remotely by means of telecommunications technology, for example, via telephone, video call or online).

a. No
b. Yes

81. **What types of telehealth/telemedicine services have you used?**

Check all that apply

- ☐ Appointments with a health professional using video conferencing
- ☐ Appointments with a health professional using telephone consultations
- ☐ Web-based treatment programs (e.g. Spinal Cord Injuries Australia (SCIA) NeuroMoves)
- ☐ Others, please specify ☐...……….
- ☐ None

82. **Who was the healthcare provider you last visited via telehealth/telemedicine? (Please consider only your most recent visit)**

a. Primary care physician / general practitioner
b. Spinal specialist working in a specialist spinal cord injury service/unit
c. Rehabilitation medical specialist
d. Local specialist (e.g. urologist, neurologist)
e. Nurse
f. Psychologist
g. Physiotherapist

h. Occupational therapist
i. Others, please specify ……………………………………………………..………

83. How would you rate your last telehealth visit compared to a traditional in-person healthcare visit?

a. Better
b. Just as good
c. Worse
d. Unsure

84. What are the reasons that you made you select the above answer?

Check all that apply

- ☐ Communication is not as effective as face-to-face visits
- ☐ The inability to be physically examined
- ☐ Difficulties with internet access
- ☐ Difficulties operating the telehealth technology
- ☐ Issues with obtaining prescriptions and pathology results
- ☐ Reduced confidence in doctor/health professional
- ☐ Additional burden for complex care
- ☐ Others, please specify ……………………………………………………..………

85. In the last 12 months, have you needed to access a telehealth service but could not?

a. No
b. Yes, what are the reasons that you could not access a telehealth service in the last 4 weeks?
   - ☐ Telehealth not available from general practitioner or other health professional
   - ☐ Do not have internet
   - ☐ I am not able to use the internet
   - ☐ Difficulties operating telehealth technology
   - ☐ Dislike or fear of the service
   - ☐ Appointment not available when required
   - ☐ Others, please specify ……………………………………………………..………

86. Compared to one year ago, how would you rate your health function and independence?

- ○ Much better
- ○ Somewhat better
- ○ About the same
- ○ Somewhat worse
- ○ Much worse

The next few questions ask about problems with skin breakdown that may affect your health, function and participation.

87. In the <u>last 12 months</u>, have you had an area of skin breakdown / ulcer / pressure injury?

- ○ No (please go to Question 91)
- ○ Yes, I have experienced one pressure injury / ulcer
- ○ Yes, I have experienced two or more pressure injuries / ulcers
- ○ Yes, I have experienced skin breakdown, but it was not caused by pressure (eg. burn)
- ○ Other, please specify: ………………………………………………………………………

88. How long did the area of skin breakdown / ulcer / pressure injury take to heal completely?

- ○ Less than 1 week
- ○ 1-4 weeks
- ○ 1-3 months
- ○ 3-6months
- ○ 6-12 months
- ○ More than 12 months

89. Did the skin breakdown / ulcer / pressure injury require an admission/s to hospital?

- ○ No
- ○ Yes, I spent days in hospital

90. **Do you have a skin breakdown / ulcer / pressure injury that has never completely healed or that breaks down frequently?**

- ○ No
- ○ Yes

Participation in activities

How many hours per week do you spend on the following activities. The following questions refer to a **normal week** (holidays and possible travel times are excluded), The questions refer to the **number of hours per week**.

Please tick the answer that best describes your situation.

91.		*0 Hours*	*1-8 Hours*	*9-16 Hours*	*17-24 Hours*	*25-35 Hours*	*35 Hours or More*
a.	**Paid work**<br>*All forms of paid work, excluding work for your own business*						
b.	**Unpaid work**<br>*Activities in an association, in a community centre, at school, or any other voluntary activities*						
c.	**Education**<br>*Only training or courses in the context of your paid work or for possible paid work in the future*						
d.	**Household duties**<br>*eg cooking, cleaning, shopping, caring for or supervising children, performing small works at home, gardening*						

How often have you performed the following activities **in the last 4 weeks**?

For example, if you take a walk approximately twice a week, this equals eight times in four weeks. In that case you choose the category "6-10 times".

Do not include any activity in more than one category. **Exclude** activities concerning work, school or household. **Exclude** travel times.

92.		*Never*	*1-2 Times*	*3-5 Times*	*6-10 Times*	*11-18 Times*	*19 Times or More*
a.	**Sports or other physical exercise**<br>*e.g. tennis, hand cycle cycling, fitness activities, long walks. Please note: rides to work with the hand cycle or wheelchair are not considered*						
b.	**Going out**<br>*e.g. eating out, going to a café, cinema, concerts, alone or with others*						
c.	**Day trips and other outdoor activities**<br>*e.g. shopping, attending events, going to the beach, visiting churches or mosques*						
d.	**Leisure activities at home**						
e.	**Visiting family or friends**						

f.	**Visits from family or friends**	
g.	**Contacting other by phone or computer**<br>*e.g. talking on the phone, texting, e-mailing*	
h.	**Meeting family/friends outside home**	

Society / Social integration

93. In my daily life I get very little chance to show how capable I am.

- ○ Agree strongly
- ○ Agree
- ○ Neither agree nor disagree
- ○ Disagree
- ○ Strongly disagree

94. I feel close to the people in my local area.

- ○ Agree strongly
- ○ Agree
- ○ Neither agree nor disagree
- ○ Disagree
- ○ Strongly disagree

95. When I hit a major problem at work or in attempting to return to work, I have co-operative co-workers or supervisors that help me overcome it.

- ○ Agree strongly
- ○ Agree
- ○ Neither agree nor disagree
- ○ Disagree
- ○ Strongly disagree

Please choose a number for each statement from 0 to 6 that indicates your level of agreement with each statement, where 0 indicates "Not at all" and 7 indicates "Strongly agree"

**96.**		*Not at all*<br>*0*	*1*	*2*	*3*	*4*	*5*	*A great deal*<br>*6*
	**Do you feel that people treat you with respect?**							
		Not at all 0	*1*	*2*	*3*	*4*	*5*	*Completely*<br>*6*
**97.**	**To what extent do you receive help and support from people you are close to when you need it?**							
		No control<br>*0*	*1*	*2*	*3*	*4*	*5*	*Extreme control*<br>*6*

98. During the past week how, much control does you feel that you have had over your life?	

When a traumatic injury or disease causes sudden spinal cord impairment, it can have profound effects on our lives. The following questions are from a scale designed to assess how your injury has affected your life. Listed below are four statements describing different thoughts and feelings that you may experience when you think about your injury.

Please indicate the degree to which you have these thoughts and feelings when you think about your injury on a 5-point scale with the endpoints where 0 indicates "not at all" and 4 indicates "all the time".

99.		*Not at all 0*	*1*	*2*	*3*	*All the time 4*
a.	**Most people don't understand how severe my condition is.**					
b.	**I am suffering because of someone else's negligence.**					
c.	**I just want my life back.**					
d.	**It all seems so unfair.**					

Please indicate how often the following statements apply to you.

100.		*Not true at all* 0	*Rarely true* 1	*Sometimes true* 2	*Often true* 3	*True nearly all the time* 4
a.	**I am able to adapt to change**					
b.	**I tend to bounce back after illness or hardship**					

Sleep Quality

People with a spinal cord injury commonly report problems with sleep. This final series of questions relate to your usual sleep habits during the past month only. Your answers should indicate the most accurate reply for the majority of days and nights in the past month. **Please answer all questions.**

101. **During the past month, what time are you usually in bed and ready to go to sleep at night?**

□.................................. □AM ....□PM

102. **During the past month, how long (in minutes) has it usually taken you to fall asleep each night?**

□................................ minutes

103. **During the past month, what time are you usually ready to get up in the morning?**

□.................................... □AM □PM

104. **During the past month, how many hours of actual sleep did you get at night? (This may be different than the number of hours you spent in bed.)** □................................ hours

**105.** During the past month, how often have you had trouble sleeping because you…		*Not during the past month*	*Less than once a week*	*Once or twice a week*	*Three or more times a week*
a.	**Cannot get to sleep within 30 minutes**				
b.	**Wake up in the middle of the night or early morning**				
c.	**Have to get up to use the bathroom**				
d.	**Cannot breathe comfortably**				
e.	**Cough or snore loudly**				
f.	**Feel too cold**				
g.	**Feel too hot**				
h.	**Have bad dreams**				
i.	**Have pain**				
j.	**Other reason(s), please describe:** □...				
k.	**During the past month, how often have you taken medicine to help you sleep (prescribed or "over the counter")?**				
l.	**During the past month, how often have you had trouble staying awake while driving, eating meals, or engaging in social activity?**				

106. **During the past month, how much of a problem has it been for you to keep up enough enthusiasm to get things done?**

- ○ No problem at all
- ○ Only a very slight problem
- ○ Somewhat of a problem
- ○ A very big problem

107. **During the past month, how would you rate your sleep quality overall?**

- ○ Very good
- ○ Fairly good
- ○ Fairly bad
- ○ Very bad

	No bed partner or room mate	*Partner / room mate in other room*	*Partner in same room but not same bed*	*Partner in same bed*
**108. Do you have a bed partner or roommate?**				

109. If you have a roommate or bed partner, ask them how often in the past month you have had:	*Not during the past month*	*Less than once a week*	*Once or twice a week*	*Three or more times a week*
**a. Loud snoring**				
**b. Long pauses between breaths while asleep**				
**c. Legs twitching or jerking while you sleep**				
**d. Episodes of disorientation or confusion during sleep**				
**e. Other restlessness while you sleep, please describe: ....**				

We thank you very much for participating in the InSCI survey!
