# Supplementary material for: LIVING WITH SPINAL CORD INJURY IN THE COMMUNITY OF BANGLADESH: A COMPREHENSIVE ANALYSIS USING THE ICF FRAMEWORK
Source: J Rehabil Med. 2026 Jun 23;58:44856. doi: 10.2340/jrm.v58.44856 (PMC13309837; doi:10.2340/jrm.v58.44856)

Fig. S1. Flowchart of the subject recruitment

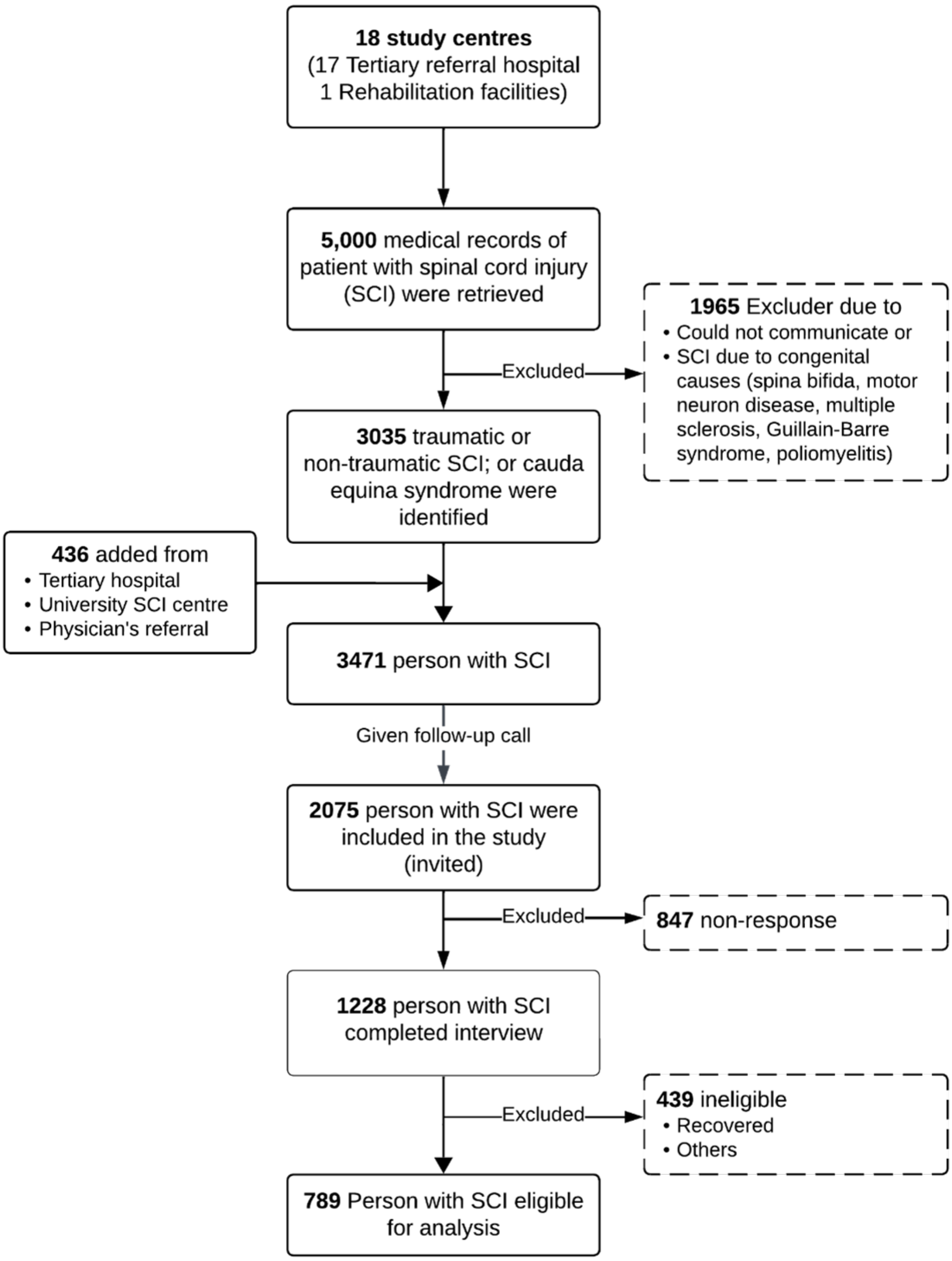

Fig. S2. Comparison of the scores of disability status (a), functional independence (b), environmental factors (c) and personal factors (c) of the person with paraplegic and tetraplegic spinal cord injury.

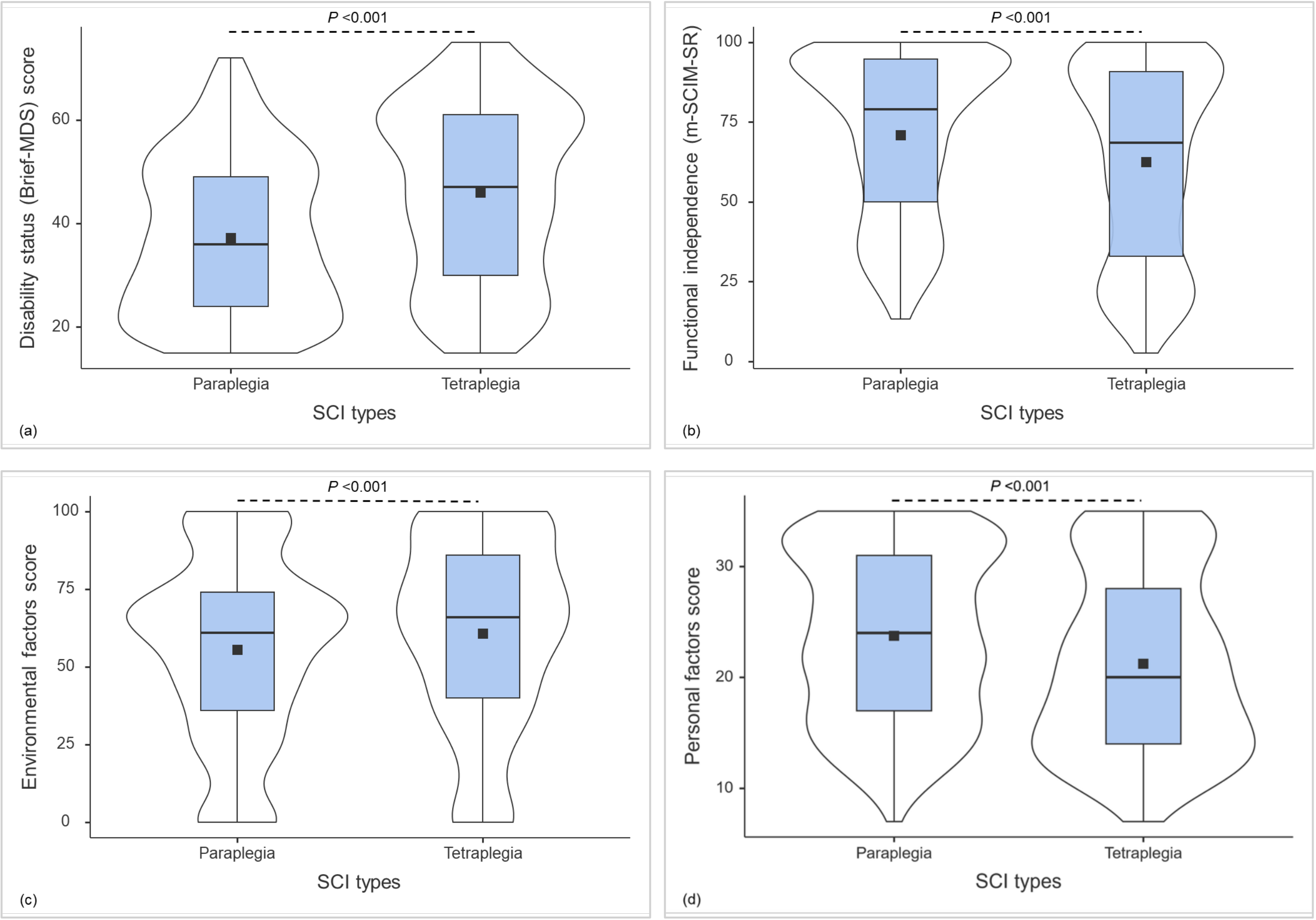

Supplement: Supplementary file 2 [file JRM-58-44856-s2.pdf]
